# Development, Analysis, and Control of Series Elastic Actuator-Driven Robot Leg

**DOI:** 10.3389/fnbot.2019.00017

**Published:** 2019-05-07

**Authors:** Chan Lee, Sehoon Oh

**Affiliations:** Motion Control Lab, Department of Robotics Engineering, Daegu Gyeongbuk Institute of Science and Technology, Daegu, South Korea

**Keywords:** biarticular actuator coordinate, series elastic actuator, rotating workspace, leg force control, impedance control

## Abstract

The mass-spring system-like behavior is a powerful analysis tool to simplify human running/locomotion and is also known as the Spring Loaded Inverted Pendulum (SLIP) model. Beyond being just an analysis tool, the SLIP model is utilized as a template for implementing human-like locomotion by using the articulated robot. Since the dynamics of the articulated robot exhibits complicated behavior when projected into the operational space of the SLIP template, various considerations are required, from the robot's mechanical design to its control and analysis. Hence, the required technologies are the realization of pure mass-spring behavior during the interaction with the ground and the robust position control capability in the operational space of the robot. This paper develops a robot leg driven by the Series Elastic Actuator (SEA), which is a suitable actuator system for interacting with the environment, such as the ground. A robust hybrid control method is developed for the SEA-driven robot leg to achieve the required technologies. Furthermore, the developed robot leg has biarticular coordination, which is a human-inspired design that can effectively transmit the actuator torque to the operational space. This paper also employs Rotating Workspace (RW), which specializes in the control of the biarticulated robots. Various experiments are conducted to verify the performance of the developed robot leg with the control methodology.

## 1. Introduction

Humans and animals' dynamic walking has been attracting many engineers' and scientists' attention, and the dynamic walking of a robot itself has been a very prominent research topic for robotic engineers.

Researches on dynamic walking can be categorized into two groups: an engineering approach and a scientific approach. The engineering approach aims to build legged robots and to find a control algorithm that can realize the dynamic walking of the robot walk. Various research aiming to build biped robots and quadruped robots (Boaventura et al., 2013; Englsberger et al., 2014; Hutter et al., 2016; Kuindersma et al., 2016; Jung et al., 2018) is considered to be within this approach. Walking algorithm theories such as Zero Moment Point stabilization are also categorized into this research approach, whereas the scientific approach attempts to analyze actual human walking and then derive a walking dynamic model (Seyfarth et al., 2002; Geyer et al., 2006). Medical researchers, physical therapists and even zoologists are becoming more involved in this research (Farley et al., 1993; Ferris et al., 1998; Zajac et al., 2002, 2003). Moreover, the advances in motion measurement technologies boost the demand for better algorithms of these approaches. There also are attempts to integrate the two approaches—the realization of dynamic walking using an actual legged robot based on dynamic characteristics.

The Spring Loaded Inverted Pendulum (SLIP) model is a promising dynamic model based on a scientific approach that can explain humans' dynamic walking and running in a simple formulation (Poulakakis and Grizzle, 2009), which is different from the complicated engineering approach. In the SLIP model, it is assumed that a (robotic) leg behaves as a pure spring. Still, building an actual robot leg that can exhibit the pure dynamic characteristics of a spring is challenging (Oh and Kong, 2014). Most recent studies have focused on ‘utilizing SLIP dynamics for locomotion/hopping’ after the work of Raibert (1986). Raibert has developed a mechanical leg design that is a combination of a linear actuator for the interaction and a rotary actuator for the leg swing motion. This mechanism is intuitively matched to the motion of SLIP dynamics, thus enabling a successful SLIP-based locomotion. The other straightforward mechanical design that has been designed is called ‘clock-actuated SLIP’ (Altendorfer et al., 2001; Seipel and Holmes, 2007). The mechanism also matches motions the mechanical characteristics of the SLIP dynamics. Even though the mechanisms have successfully realized SLIP-based running/locomotion, they are still different from the real human- or animal-like leg. Therefore, these approaches can be utilized only for their respective application.

Furthermore, some researchers have intensively analyzed this problem and came up with control algorithms that can address the realization of dynamic motion based on robot dynamics (Yamaguchi et al., 1999; Khatib et al., 2008; Tsagarakis et al., 2013; Koolen et al., 2016; Oehlke et al., 2018). For instance, Hutter et al. (2010) claimed that the problem is caused by the inherent dynamics of the articulated robot in the operational space and proposed an operational space dynamics control. However, this research is still in a bottleneck because the method does not utilize any feature of the articulated leg or the specific workspace. Therefore, the method needs improvement of the dynamic modeling, with additional complexity coming from the modeling based on the Cartesian workspace. However, it can be said that there is still a large discrepancy between the two approaches. Therefore, the previously proposed walking dynamics still cannot be applied to the actual robot, because the dynamic behavior of the actual robot is quite different from the theoretical model, especially during fast and dynamic motions. Moreover, there is no recent significant finding after (Hutter et al., 2010) to provide the solution for enabling pure SLIP dynamics of the articulated leg.

The other significant issues due to the discrepancy are the difference of the dynamic model disturbed by external forces and the nominal dynamic model, and as a result, it is more difficult to control. Specifically, in the dynamic reaction of a robot walking, the ground reaction force affects the robot continuously. Hence, force control is applied to the leg to render the dynamic response of a robot leg against external force. Nowadays, many humanoid robots have employed a force/torque control platform (Englsberger et al., 2014; Hyon et al., 2017). However, force control of a robot leg has various challenges such as friction and noise in the force sensors. To solve this problem, a robot leg that is fully driven by two Series Elastic Actuators (SEAs) is developed in this research.

Since SEAs enable high-performance force control, the proposed robot leg exploits this high-performance force control to realize ideal SLIP dynamics. There are remarkable studies which equip SEAs for the biped robot called “ATRIAS” (Rezazadeh et al., 2015; Martin et al., 2017). However, the main difference with this paper is that ATRIAS utilizes the SEAs as a velocity source to regulate the ground reaction force under the SLIP-based high-level controls and switches the control method depending on the gait phase (flight/stance). In this paper, the SEAs are focused on a realization of pure SLIP dynamics based on their force controllability.

In addition to the employment of SEAs, the introduction of the biarticular actuation mechanism is applied to facilitate the realization of SLIP dynamics of the articulated robot leg. The biarticular actuation is known for its specific functionalities and roles in human motions (van Ingen Schenau et al., 1987; Zajac et al., 2002, 2003). Also, many biomimetic robots have employed this mechanism for jumping and running (Hurst et al., 2010; Niiyama et al., 2010). Taking into consideration this advantage of the biarticular actuation mechanism, the proposed robot leg is designed based on this mechanism. The dynamic analysis of joint-space motion is conducted based on the biarticular actuator coordination.

The approaches above are considered as mechanical contributions to realizing SLIP dynamics using an articulated robot. Contribution from software can be seen in the development of Hybrid Control (HC). HC is a combination of impedance control for the realization of spring dynamics and position control for attack angle control of the SLIP dynamics. To implement this controller, suitable operational space—which is called Rotating Workspace (RW)—is employed (Oh and Kong, 2014). The dynamic behavior of the developed robot leg in the RW is analyzed to improve the performance of HC. This paper proposes a serial and systematic procedure to achieve SLIP dynamics by fully utilizing the advantage of the biarticular mechanism as well as the employment of SEAs and HC in RW.

## 2. Materials and Methods

### 2.1. Design of Series Elastic Actuator-Driven Robot Leg

**Figure 1:**
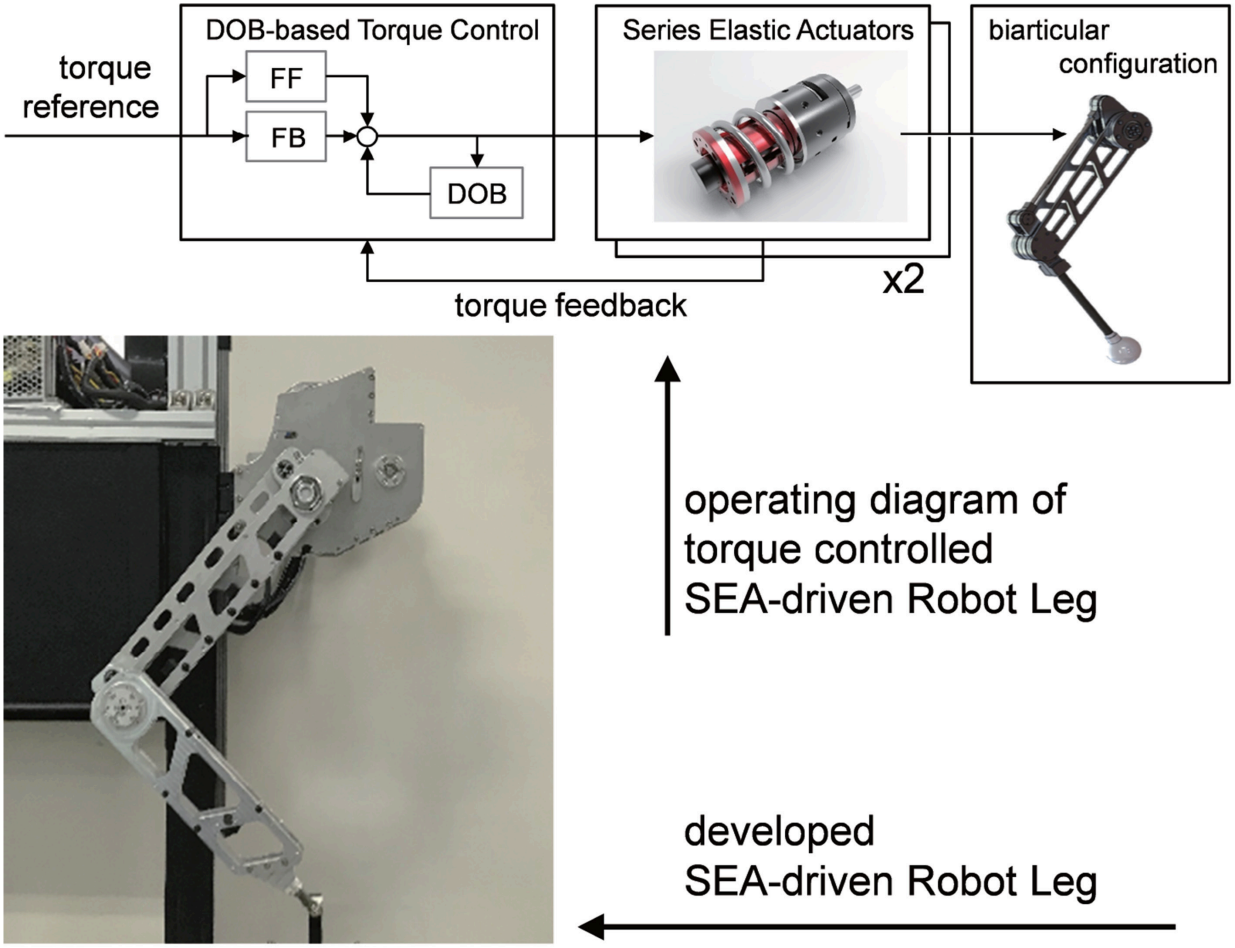
Developed series elastic actuator-driven biarticular robot leg including force controller.

Figure 1 illustrates the force-controllable robot leg which is developed in this research. The development of the robot leg is 2-fold: the introduction of the biarticular actuator mechanism and the application of the Series Elastic Actuator. The following subsections provide the advantages of these design components and methodologies of force control.

#### 2.1.1. Design Motivation of Bio-inspired Robot Leg Using Biarticular Actuator Mechanism

**Figure 2:**
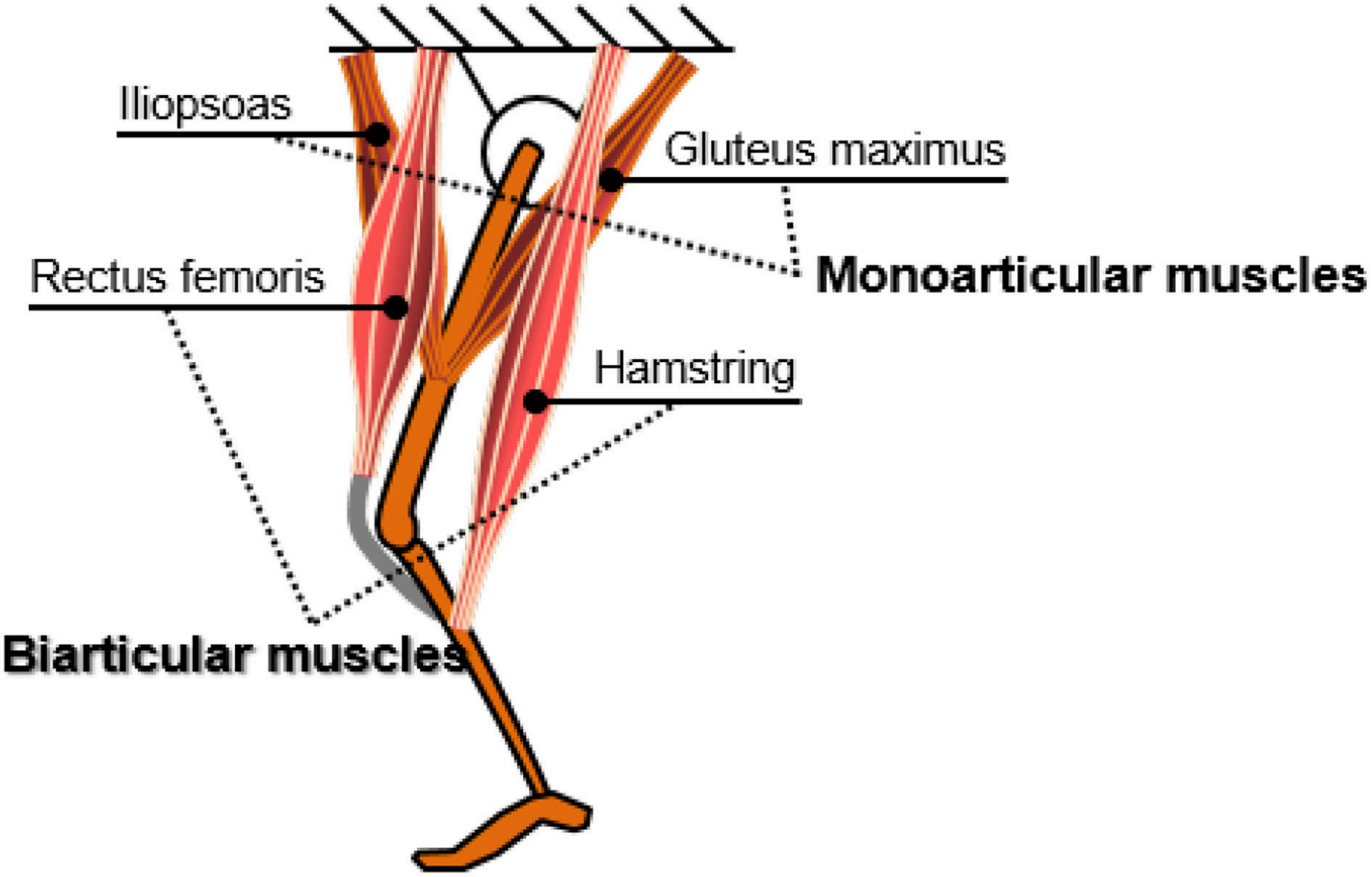
Representative biarticular muscles in the human lower extremity.

In the musculoskeletal system, biarticular muscles are muscles that cross two joints to generate torque at both joints. Most of the human muscles that generate a large amount of force or power are usually biarticular muscles, such as the rectus femoris at the front thighs, and the hamstring at the back thighs as shown in Figure 2.

In the human locomotion mechanism, biarticular muscles play a vital role to link the movement of muscles in the limb (Zajac et al., 2002, 2003). When a joint is locked by the co-contraction of a pair of monoarticular muscles, the associated biarticular muscles act as powerful monoarticular muscles that actuate the whole extremity. Also, the length change of the biarticular muscles is much smaller than that of the monoarticular muscles for generating normal human motion (van Ingen Schenau et al., 1987).

Consequently, the biarticular muscle mechanism enables the high efficiency and performance of the human lower extremity movement. This high efficiency has been theoretically (Oh et al., 2011) analyzed and evaluated through experiments (Choi et al., 2016; Roozing et al., 2018).

#### 2.1.2. Implementation of Biarticular Actuator Mechanism

**Figure 3:**
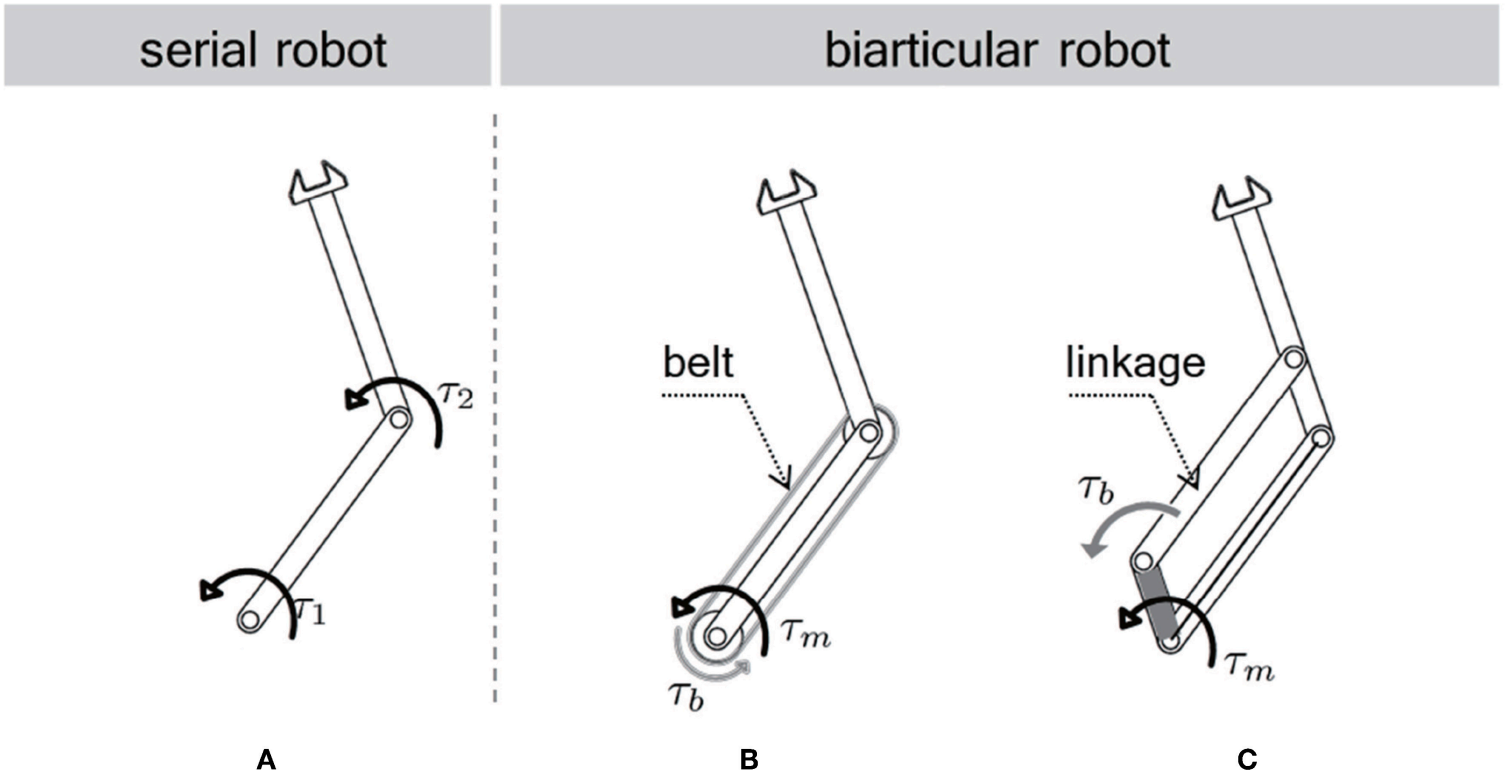
Various configurations of a two link robot. **(A)** series configuration, **(B)** biarticular configuration (wire/belt-driven), **(C)** biarticular configuration (linkage-driven).

Muscle coordination is known to play an essential role in various multi-joint human motions (Zajac, 1993). Similar to this concept, the actuator coordination of a robot is also to be well-designed to enhance the performance of actuators in a system. Figure 3 illustrates possible actuator coordinations to configure a two-link robot. Figure 3A is the conventional serial type two link robot, each joint of which is equipped with an actuator. Oh et al. (2011) has shown the inefficiency of this actuator coordinate in terms of torque/force generation. Moreover, the actuator is located on each link in this configuration which imposes the weight of the actuator on every link. It is also noticeable that this configuration needs to utilize the relative joint coordinates to describe its motion.

Figures 3B,C illustrates the biarticular actuator coordination in a two-link robot, where the belt/wire in Figure 3B and the linkage in Figure 3C are utilized to transmit the torque of one actuator in the proximal joint to the distal link. This transmission enables the biarticular actuator coordination in a robotic system (Choi et al., 2016; Roozing et al., 2016). As this mechanism can locate the actuators on the proximal joint, the weight of the distal link is reduced, which is considered as another advantage.

This research adopts the link mechanism in Figure 3C to employ the biarticular actuator coordination as well as to guarantee large force transmission along with robustness against an impact from the environment.

**Figure 4:**
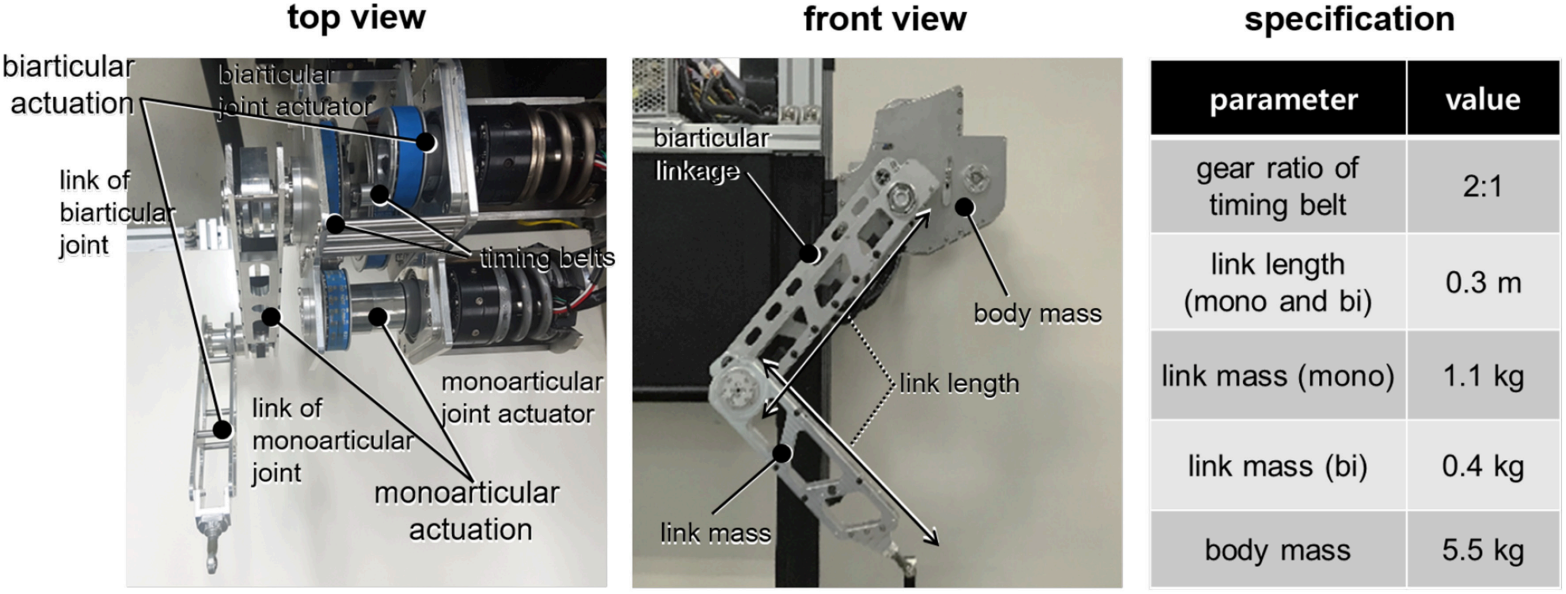
Details of the developed SEA-driven biarticular robot leg: top view, front view and specifications of the robot.

Figure 4 shows the details of the developed SEA-driven biarticular robot leg. Two SEAs are connected on the body to provide mono- and biarticular torques. The torques are transmitted to the links through the timing belts, which have a gear ratio of 2:1. For simplicity of the dynamics, both mono- and biarticular-side links are designed to be equal to 0.3 m. The specifications of the parameters are summarized in the table on the right-hand side of Figure 4.

#### 2.1.3. Actuator Design: Transmission Force Sensing Type Series Elastic Actuator

SEA of Pratt and Williamson (1995) utilizes a spring as a part of its transmission to measure and control the interacting force between the motor and the load. As explained in section 1, SEA is employed as the actuator system in this research since it can provide high-fidelity torque/force while it is robust against the impact due to its inherent compliance.

There are various types of SEA configurations (Lee et al., 2017) such as the direct force sensing type (FSEA) (Pratt and Williamson, 1995), the reaction force sensing type (RFSEA) (Paine et al., 2014; Park et al., 2018) and the transmission force sensing SEA (TFSEA), Lee and Oh (2016). TFSEA is utilized as the actuators for the proposed robot leg.

**Figure 5:**
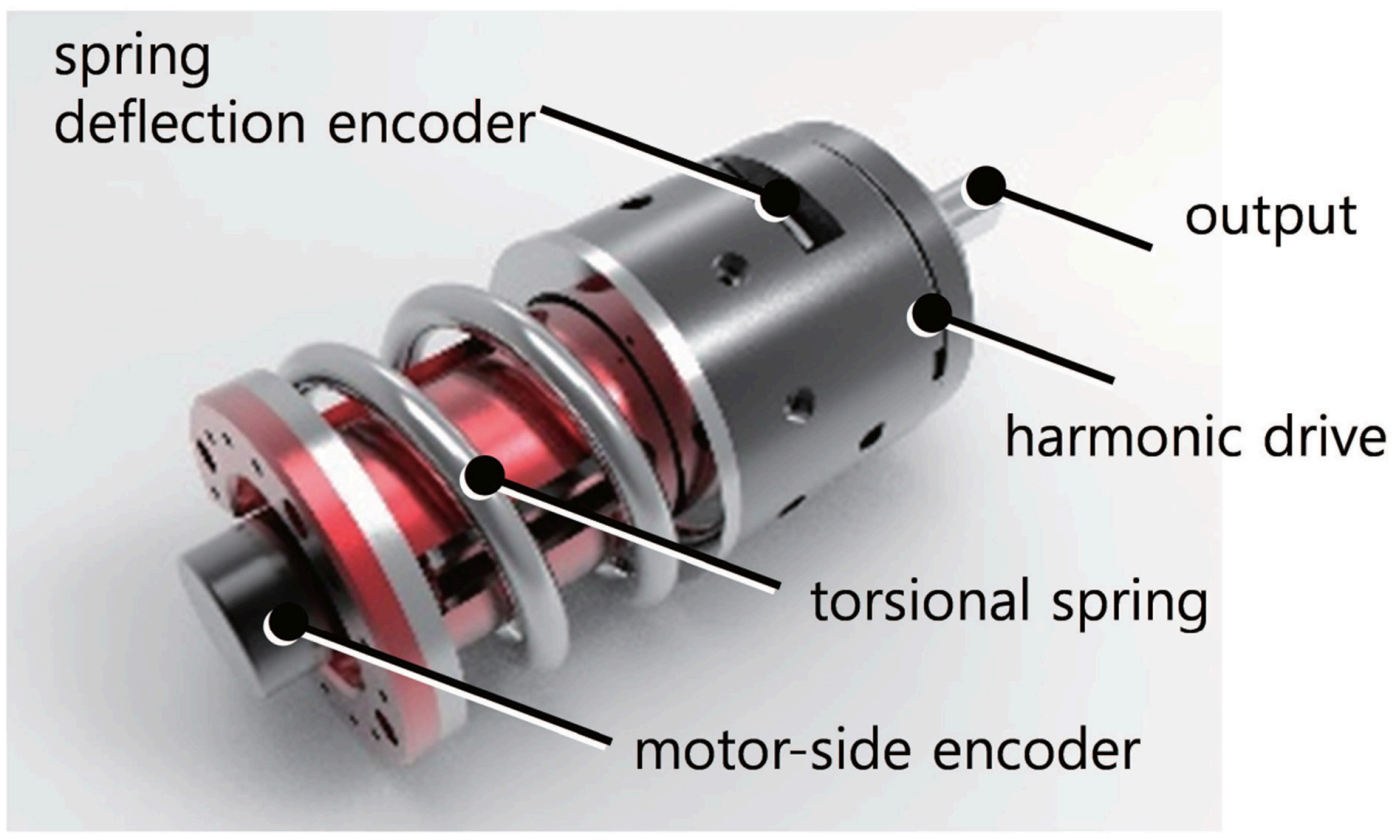
Developed transmission force sensing type series elastic actuator (TFSEA).

Figure 5 shows the TFSEA utilized in this research, where the spring is located between the ring gear of the planetary gear and the ground. This configuration allows the measurement of the absolute transmission force by using a single encoder and the robustness against the dead zone caused by gear backlash. The details of the configuration of TFSEA are described in Lee and Oh (2016).

The SEA in Figure 5 developed for the robot leg consists of a current/torque-controlled BLDC motor with a servo driver (Maxon motor, EC 4-pole 48V and ESCON70/10), a harmonic gear (Harmonic Drive®, CSF-11), a torsional spring (custom-made), and two encoders (incremental type motor-side encoder is 5,000 count/turn resolution and absolute type spring deflection encoder is 19-bit resolution). The maximum continuous torque of the SEA is 55.2 Nm (peak: 686 Nm), and the maximum permissible velocity is 80 rpm. The stiffness of the spring is 128 Nm/rad, which leads to a high force sensing resolution of 0.3835 mN-m/tick with a 19-bit absolute encoder. Note that these specifications including output stiffness are obtained in the biarticular joint coordinates after timing belts and gears as shown in Figure 4.

#### 2.1.4. Robust Torque Control for the Developed SEA to Provide Ideal Torque Source

To achieve high-performance force control of the cPEA in the proposed robotic leg, model-based force control developed by Oh and Kong (2017) is applied in this research.

**Figure 6:**
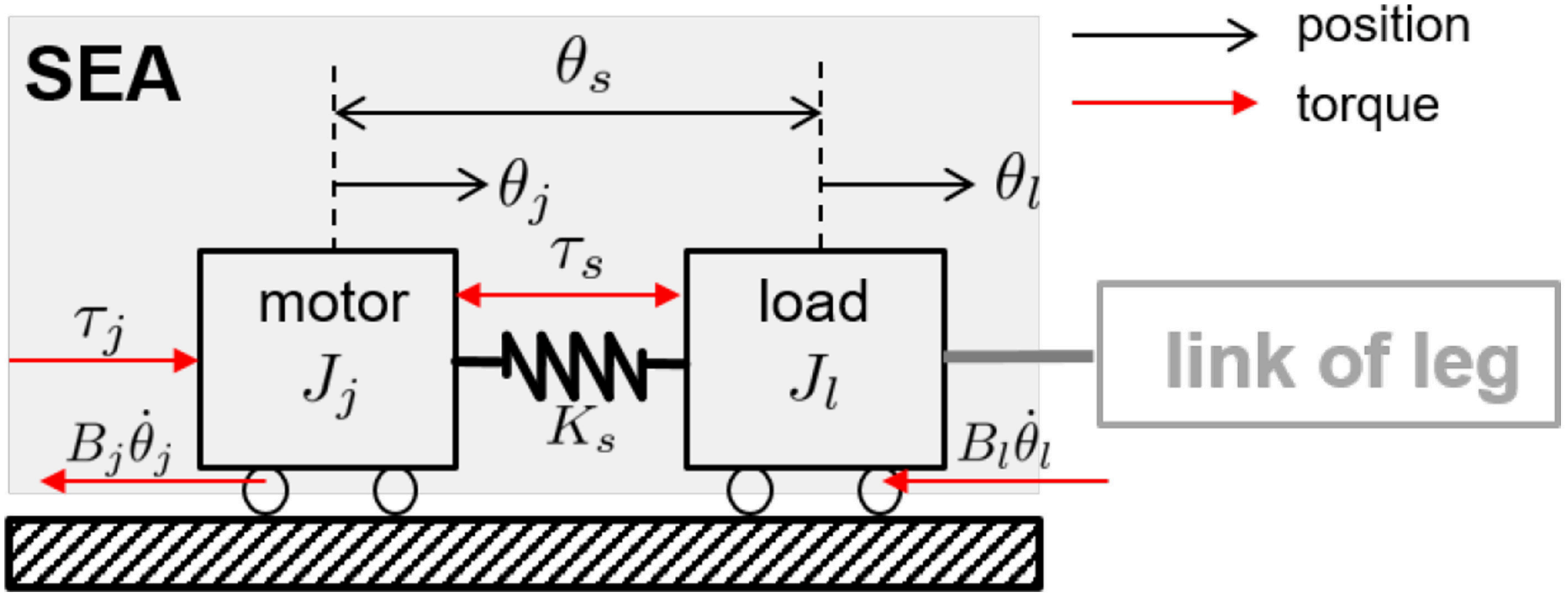
Free body diagram of the SEA. The black-colored arrows indicate motor, load position and spring deflections, and the red-colored arrows denote motor, spring forces and friction force.

To design the model-based force control, the general dynamics of the SEA is derived from the free-body diagram in Figure 6 as follows:

(1) $$\begin{array}{l} J_{m}{\ddot{\theta }}_{j}=\tau_{j}-\tau_{s}-B_{j}{\dot{\theta }}_{j},\\ {\;J}_{l}{\ddot{\theta }}_{l}=\tau_{s}-B_{l}{\dot{\theta }}_{l},\\ {\;\tau }_{s}=K_{s}\theta_{s}, \end{array}$$

where *J*_*j*_ and *J*_*l*_ are the moment of inertias of the motor and the load, *B*_*j*_ and *B*_*l*_ are the joint damping coefficients of the motor and the load, and *θ*_*j*_ and *θ*_*l*_ are the joint position of the motor and the load. *θ*_*s*_ is the spring deflection. *τ*_*j*_ is the torque generated by the motor and *τ*_*s*_ is the torque transmitted through the spring. *K*_*s*_ is the spring stiffness.

From the dynamics of the SEA in Equation (1), the transfer function from the motor torque *τ*_*j*_ to the SEA transmission torque *τ*_*s*_ can be obtained as follows.

(2) $$\begin{array}{l} P_{s}\left(s\right)=\frac{\tau_{s}}{\tau_{j}}=\frac{K_{s}\left(J_{l}s+B_{l}\right)}{\left(J_{l}s+B_{l}\right)\left(J_{j}s^{2}+B_{j}s\right)+K_{s}\left\{\left(J_{l}+J_{j}\right)s+\left(B_{l}+B_{j}\right)\right\}} \end{array}$$

The actuator dynamics of SEA is basically linear. However, the value of the load-side moment of inertia *J*_*l*_ is subjected to varying joint angle conditions, particularly in a multi-link robot like the proposed robotic leg. Therefore, a robust torque controller is required to provide an ideal torque source to the proposed robotic leg regardless of the joint angle.

**Figure 7:**
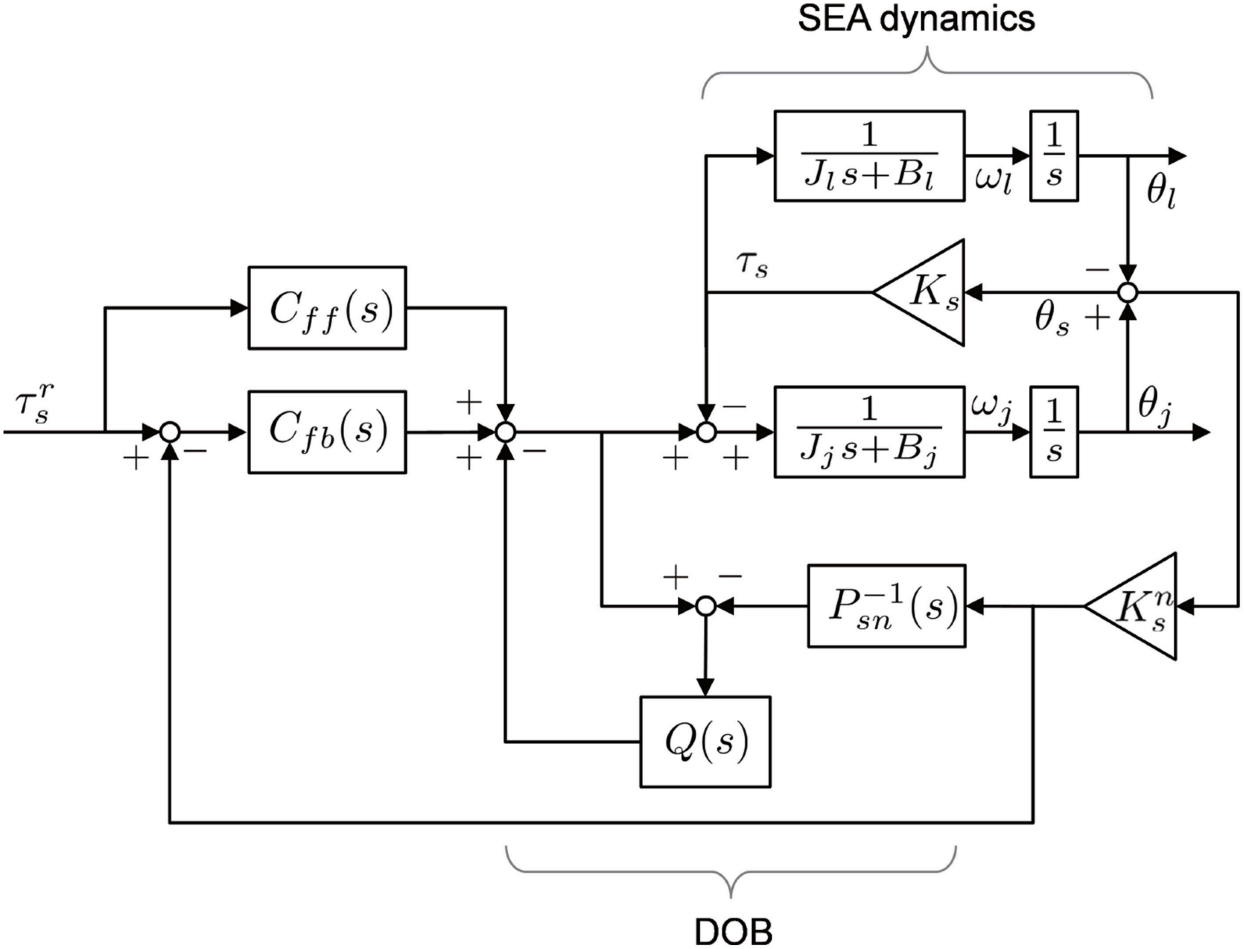
Block diagram of SEA dynamics with Disturbance Observer-based robust torque control.

To that end, a Disturbance Observer (DOB)-based robust torque controller is applied for the torque control of two SEAs. The overall control scheme of the SEA torque control is illustrated in Figure 7. In Figure 7, $P_{sn}^{-1}\left(s\right)$ indicates the inverse model of the nominal transmission dynamics in Equation (2), and *Q*(*s*) is the second-order Q-filter of DOB. *C*_*fb*_(*s*) and *C*_*ff*_(*s*) are the feedback and feedforward controller, respectively.

DOB can nominalize the dynamics of SEA in a specific frequency bandwidth determined by the Q-filter, leading to a robust force control against varying load conditions. *C*_*ff*_(*s*) is designed as the inverse model of the nominalized SEA dynamics, with a low-pass filter that can enhance fast-tracking performance.

The performance of the torque control algorithm is verified using the closed-loop frequency response measurements. To investigate the robustness of the torque control, these experiments were conducted with two different load conditions: with free-load, which means no additional load is attached to the SEA, and with the fixed-load environment. To analyze the performance of the frequency responses in these load conditions, a chirp-type excitation signal is applied with frequencies of 0.1–100 Hz. The output torques of SEA are measured by a torque sensor, and the experiments were conducted ten times to calculate the average frequency responses.

**Figure 8:**
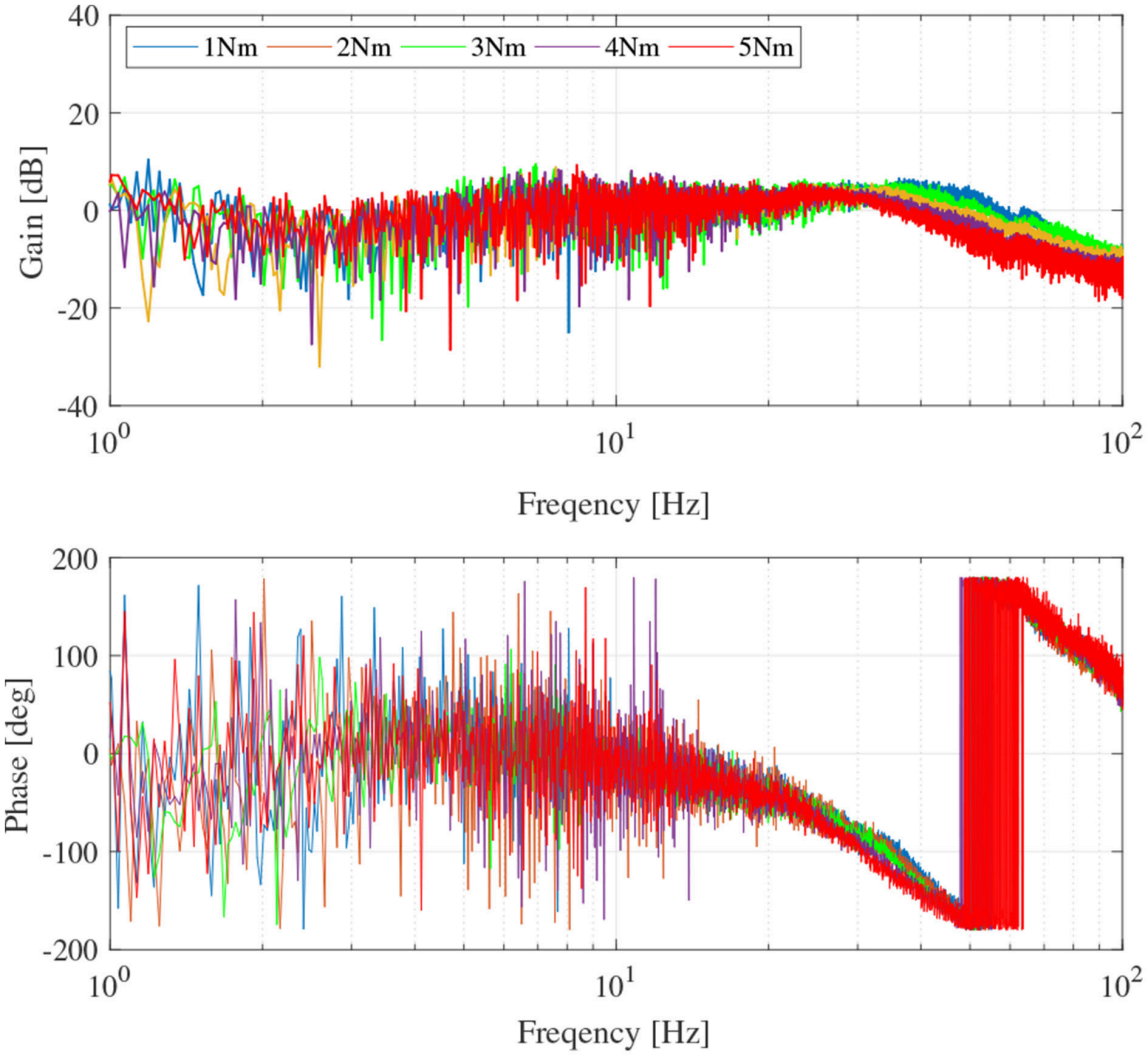
Large force bandwidth characteristics of torque-controlled SEA.

Note that the performance of the closed-loop response of the SEA torque control depends on the magnitude of the torque reference. This concept is called Large Force Bandwidth (LFB) (Hutter et al., 2016). Figure 8 shows the characteristics of the torque-controlled SEA in this paper as well as the bandwidth variance regarding the magnitude values of the reference. Moreover, 5 Nm (red-colored) is the critical magnitude value before the safety protection of the motor driver is enabled (safety stop has occurred at the high-frequency region with 6 Nm magnitude reference). Therefore, 5 Nm is chosen as the magnitude of the reference torque for the evaluation of the robustness of the SEA torque control.

**Figure 9:**
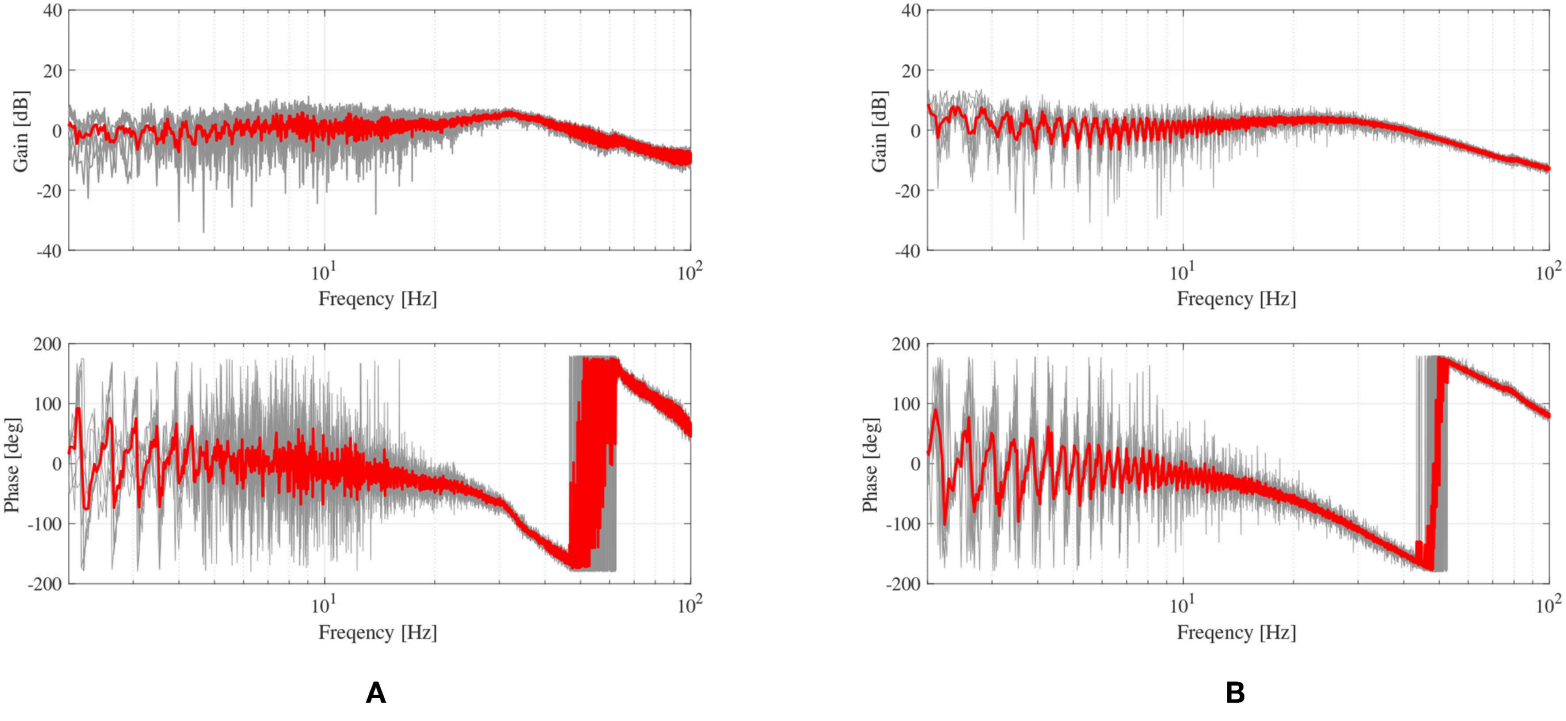
Frequency responses of the proposed force control of the TFSEA. **(A)** force control with free load condition, and **(B)** with locked-load condition.

The experimental results for the robustness evaluation are shown in Figure 9, where the plots in Figure 9A are the responses with the free-load, and the plots in Figure 9B are the responses with the fixed-load. The gray-colored lines are the results of ten repetitions, and the red-colored lines are the averaged response. The responses are matched with second-order low-pass filters: $\frac{1}{0.005^{2}s^{2}+0.004s+1}$ and $\frac{1}{0.0045^{2}s^{2}+0.003s+1}$. The bandwidths are 32 and 35 Hz, respectively, which can be calculated from the filter models.

The results verify that the SEA with the model-based force control can provide high-fidelity force generation with small performance variation (8.57%). It can also be validated that the closed-loop frequency bandwidth of the force generation is up to 32 Hz regardless of the load condition.

### 2.2. Theoretical Analysis of Biarticular Actuator Coordination

For the realization of SLIP dynamics using SEAs, the kinetic characteristics of the robot are analyzed in the joint space. Since the developed robot incorporates the biarticular actuator coordination, the kinematic and dynamic characteristics should be formulated in this coordination.

#### 2.2.1. Kinematics and Statics Analysis of Biarticular Coordination

**Figure 10:**
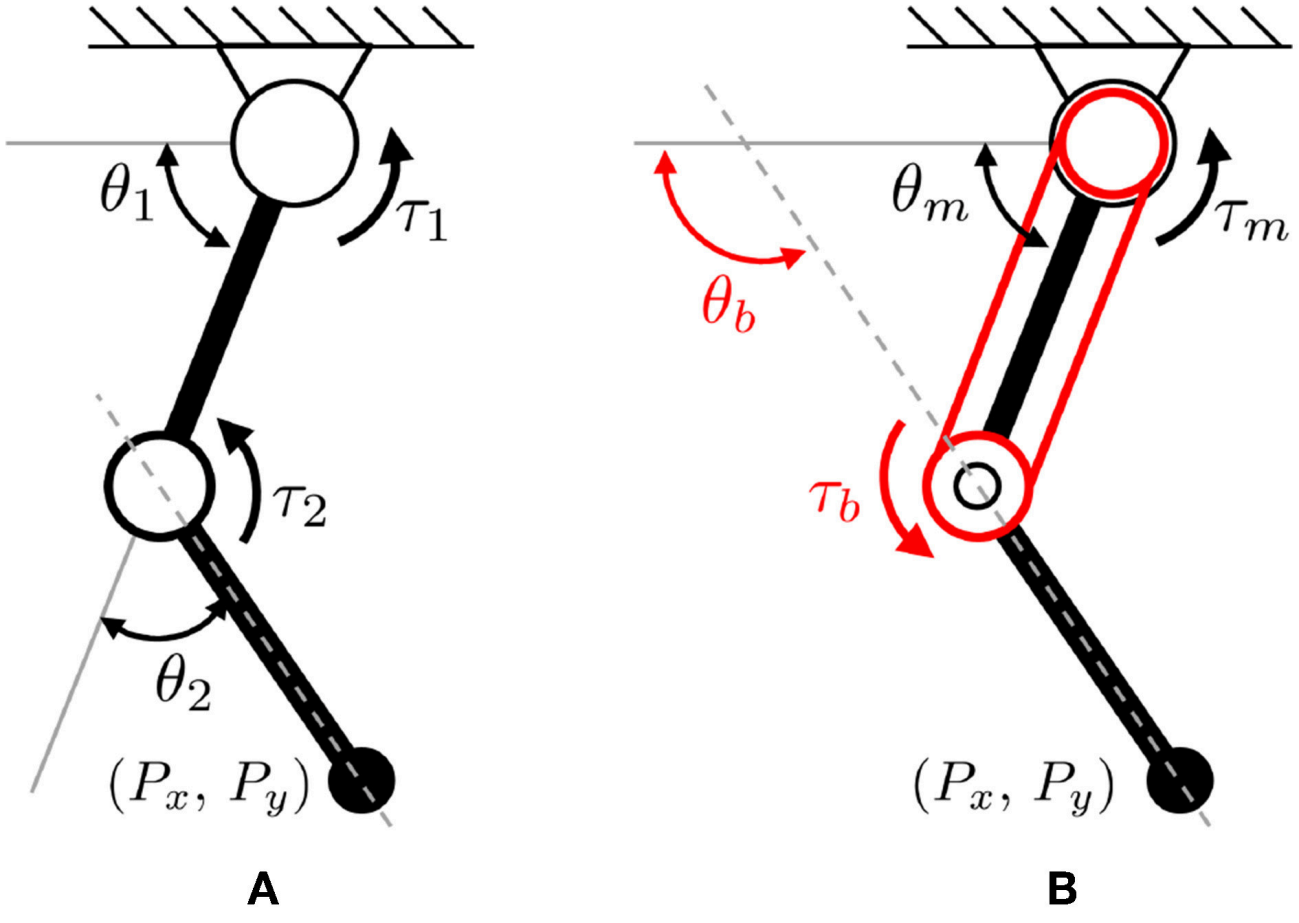
Basic configuration of a two-link system with two types of actuator coordination. **(A)** conventional serial actuator coordinates, and **(B)** biarticular actuator coordinates.

The basic actuator configurations of a two-link robot are illustrated in Figure 10 where Figure 10A is the conventional serial link with two independent (monoarticular) actuators (which generate torques *τ*_1_ and *τ*_2_), and Figure 10B is the proposed configuration with one monoarticular actuator and one biarticular actuator (which generate torques *τ*_*m*_ and *τ*_*b*_).

The kinematics of the conventional serial robot configuration shown in Figure 10A is given as

(3) $$\begin{array}{l} P_{x}=l\cos\theta_{1}+l\cos\left(\theta_{1}+\theta_{2}\right)\\ P_{y}=l\sin\theta_{1}+l\sin\left(\theta_{1}+\theta_{2}\right), \end{array}$$

where *P*_*x*_ and *P*_*y*_ are the end effector position in the Cartesian coordinate system, and *θ*_1_ and *θ*_2_ represent the angles of two joints. *l* is the length of links. Note that the length of the first and second links are the same as *l* in this paper.

On the other hand, the kinematics of the biarticular actuator-coordinated link (in Figure 10B) is described using the monoarticular joint angle *θ*_*m*_ and the absolute biarticular joint angle *θ*_*b*_, which are related to the conventional joint angles *θ*_1_ and *θ*_2_ as follows.

(4) $$\begin{array}{l} {\;\theta }_{m}=\theta_{1},\\ \theta_{b.r}=\theta_{2},\\ {\;\theta }_{b}=\theta_{1}+\theta_{2}=\theta_{m}+\theta_{b.r} \end{array}$$

where *θ*_*b*.*r*_ is relative angle between the links of the monoarticular and biarticular joints.

Based on this relationship, the end effector position *P*_*x*_ and *P*_*y*_ can be re-described using the biarticular coordination *θ*_*m*_ and *θ*_*b*_ as follows.

(5) $$\begin{array}{l} P_{x}=l\cos\theta_{m}+l\cos\theta_{b}\\ P_{y}=l\sin\theta_{m}+l\sin\theta_{b} \end{array}$$

The Jacobian matrix for the biarticular coordination can be derived by utilizing the partial differentiation of Equation (5) as follows:

(6) $$\begin{array}{l} \left[\begin{array}{c} {\dot{P}}_{x}\\ {\dot{P}}_{y} \end{array}\right]=l\left[\begin{array}{cc} -\sin\theta_{m} & -\sin\theta_{b}\\ \cos\theta_{m} & \cos\theta_{b} \end{array}\right]\left[\begin{array}{c} {\dot{\theta }}_{m}\\ {\dot{\theta }}_{b} \end{array}\right]=J_{b}\left[\begin{array}{c} {\dot{\theta }}_{m}\\ {\dot{\theta }}_{b} \end{array}\right], \end{array}$$

where ***J***_*b*_ is Jacobian for the biarticular coordination. The statics of the biarticular coordination can also be derived utilizing this Jacobian ***J***_*b*_ as follows.

(7) $$\begin{array}{l} \left[\begin{array}{c} \tau_{m}\\ \tau_{b} \end{array}\right]=J_{b}^{T}\left[\begin{array}{c} F_{x}\\ F_{y} \end{array}\right]=l\left[\begin{array}{cc} -\sin\theta_{m} & \cos\theta_{m}\\ -\sin\theta_{b} & \cos\theta_{b} \end{array}\right]\left[\begin{array}{c} F_{x}\\ F_{y} \end{array}\right] \end{array}$$

This statics can be reorganized as follows, which verifies that the biarticular torque *τ*_*b*_ works on the first joint and the second joint at the same time.

(8) $$\begin{array}{l} \left[\begin{array}{c} \tau_{1}\\ \tau_{2} \end{array}\right]=\left[\begin{array}{cc} 1 & 1\\ 0 & 1 \end{array}\right]\left[\begin{array}{c} \tau_{m}\\ \tau_{b} \end{array}\right] \end{array}$$

#### 2.2.2. Dynamics of Two-Link Robot With Biarticular Actuator Coordination

Equation (9) describes the dynamics of a two-link robot with the conventional actuator coordination, which has two independent torque inputs *τ*_1_ and *τ*_2_ shown in Figure 10A, where ${\ddot{\theta }}_{1}$ and ${\ddot{\theta }}_{2}$ are the accelerations of two joints. *g* is the acceleration of gravity, *l*_*ic*_ is the distance from the center of a joint *i* to the center of the mass point of the link *i*, *m*_*i*_ is the weight of the link *i* and *I*_*i*_ is the moment of inertia.

(9) $$\begin{array}{l} \left[\begin{array}{cc} I_{1}+I_{2}+m_{2}l^{2}+2m_{2}l_{2c}l\cos\theta_{2} & I_{2}+m_{2}l_{2c}l\cos\theta_{2}\\ I_{2}+m_{2}l_{2c}l\cos\theta_{2} & I_{2} \end{array}\right]\left[\begin{array}{c} {\ddot{\theta }}_{1}\\ {\ddot{\theta }}_{2} \end{array}\right]\\ \;+\left[\begin{array}{c} -m_{2}l_{2c}l\sin\theta_{2}\left({\dot{\theta }}_{2}^{2}+2{\dot{\theta }}_{1}{\dot{\theta }}_{2}\right)\\ m_{2}l_{2c}l\sin\theta_{2}{\dot{\theta }}_{1}^{2} \end{array}\right]\\ \;+\left[\begin{array}{c} g\left(m_{1}l_{1c}+m_{2}l\right)\cos\theta_{1}+gm_{2}l_{2c}\cos\left(\theta_{1}+\theta_{2}\right)\\ gm_{2}l_{2c}\cos\left(\theta_{1}+\theta_{2}\right) \end{array}\right]=\left[\begin{array}{c} \tau_{1}\\ \tau_{2} \end{array}\right] \end{array}$$

This dynamics description can be rewritten utilizing the biarticular coordinates with *θ*_*m*_, *θ*_*b*_, *τ*_*m*_ and *τ*_*b*_. Equation (10) represents the relationship between the biarticular coordinates and the conventional coordinates *θ*_1_, *θ*_2_, *τ*_1_ and *τ*_2_.

(10) $$\begin{array}{l} \left[\begin{array}{c} \theta_{1}\\ \theta_{2} \end{array}\right]=\left[\begin{array}{cc} 1 & 0\\ -1 & 1 \end{array}\right]\left[\begin{array}{c} \theta_{m}\\ \theta_{b} \end{array}\right],\left[\begin{array}{c} \tau_{m}\\ \tau_{b} \end{array}\right]=\left[\begin{array}{cc} 1 & -1\\ 0 & 1 \end{array}\right]\left[\begin{array}{c} \tau_{1}\\ \tau_{2} \end{array}\right] \end{array}$$

Utilizing this relationship, the dynamics description in Equation (9) can be rewritten as in Equation (11), which is the dynamics description in the biarticular coordinates.

(11) $$\begin{array}{l} \left[\begin{array}{c} \tau_{m}\\ \tau_{b} \end{array}\right]=\left[\begin{array}{cc} I_{m} & I_{c}\left(\theta_{b.r}\right)\\ I_{c}\left(\theta_{b.r}\right) & I_{b} \end{array}\right]\left[\begin{array}{c} {\ddot{\theta }}_{m}\\ {\ddot{\theta }}_{b} \end{array}\right]\\ \;+\left[\begin{array}{c} \tau_{m.c}\left(\theta_{b.r},{\dot{\theta }}_{b}\right)\\ \tau_{b.c}\left(\theta_{b.r},{\dot{\theta }}_{m}\right) \end{array}\right]+\left[\begin{array}{c} \tau_{m.g}\left(\theta_{m}\right)\\ \tau_{b.g}\left(\theta_{b}\right) \end{array}\right], \end{array}$$

where the inertia is reformulated as

(12) $$\begin{array}{l} {\;I}_{m}=I_{1}+m_{2}l^{2}\\ {\;I}_{b}=I_{2}\\ I_{c}\left(\theta_{b.r}\right)=m_{2}l_{2c}l\cos\theta_{b.r}=C_{I}\cos\theta_{b.r}, \end{array}$$

Coriolis force and gravity effect in the biarticular coordinate are reorganized as follows:

(13) $$\begin{array}{l} \tau_{m.c}\left(\theta_{b.r},{\dot{\theta }}_{b}\right)=-m_{2}l_{2c}l\sin\theta_{b.r}{\dot{\theta }}_{b}^{2}=-C_{I}\sin\theta_{b.r}{\dot{\theta }}_{b}^{2},\\ \tau_{b.c}\left(\theta_{b.r},{\dot{\theta }}_{m}\right)=m_{2}l_{2c}l\sin\theta_{b.r}{\dot{\theta }}_{m}^{2}=C_{I}\sin\theta_{b.r}{\dot{\theta }}_{m}^{2}. \end{array}$$

(14) $$\begin{array}{l} \text{And}\\ \tau_{m.g}\left(\theta_{m}\right)=g\left(m_{1}l_{1c}+m_{2}l_{1}\right)\cos\theta_{m}=G_{m}\cos\theta_{m},\\ {\;\tau }_{b.g}\left(\theta_{b}\right)=gm_{2}l_{2c}\cos\theta_{b}=\frac{g}{l}C_{I}\cos\theta_{b}=G_{b}\cos\theta_{b}. \end{array}$$

Notice that Coriolis torques $\tau_{m.c}\left(\theta_{b.r},{\dot{\theta }}_{m}\right),\tau_{b.c}\left(\theta_{b.r},{\dot{\theta }}_{b}\right)$ and gravity effects *τ*_*m*.*g*_(*θ*_*m*_), *τ*_*b*.*g*_(*θ*_*b*_) consist of sole physical values. Namely, Coriolis torque *τ*_*m*.*c*_ is proportional only to ${\dot{\theta }}_{b}^{2}$, and *τ*_*b*.*c*_ is also proportional only to ${\dot{\theta }}_{m}^{2}$. Moreover, the gravity terms *τ*_*m*.*g*_ and *τ*_*b*.*g*_ are also proportional to cos *θ*_*m*_ and cos *θ*_*b*_, respectively.

Furthermore, the common coupling coefficient *C*_*I*_ appears in many equations including Coriolis torque in (13) and the gravity effect *G*_*b*_ in (14), which are basically constant coefficients. In other words, the coupling coefficient and the gravity effects can be identified in a simple way with these biarticular coordinates.

### 2.3. Bio-inspired Leg Interaction Control to Realize SLIP Dynamics Using SEA-Driven Robot Leg

**Figure 11:**
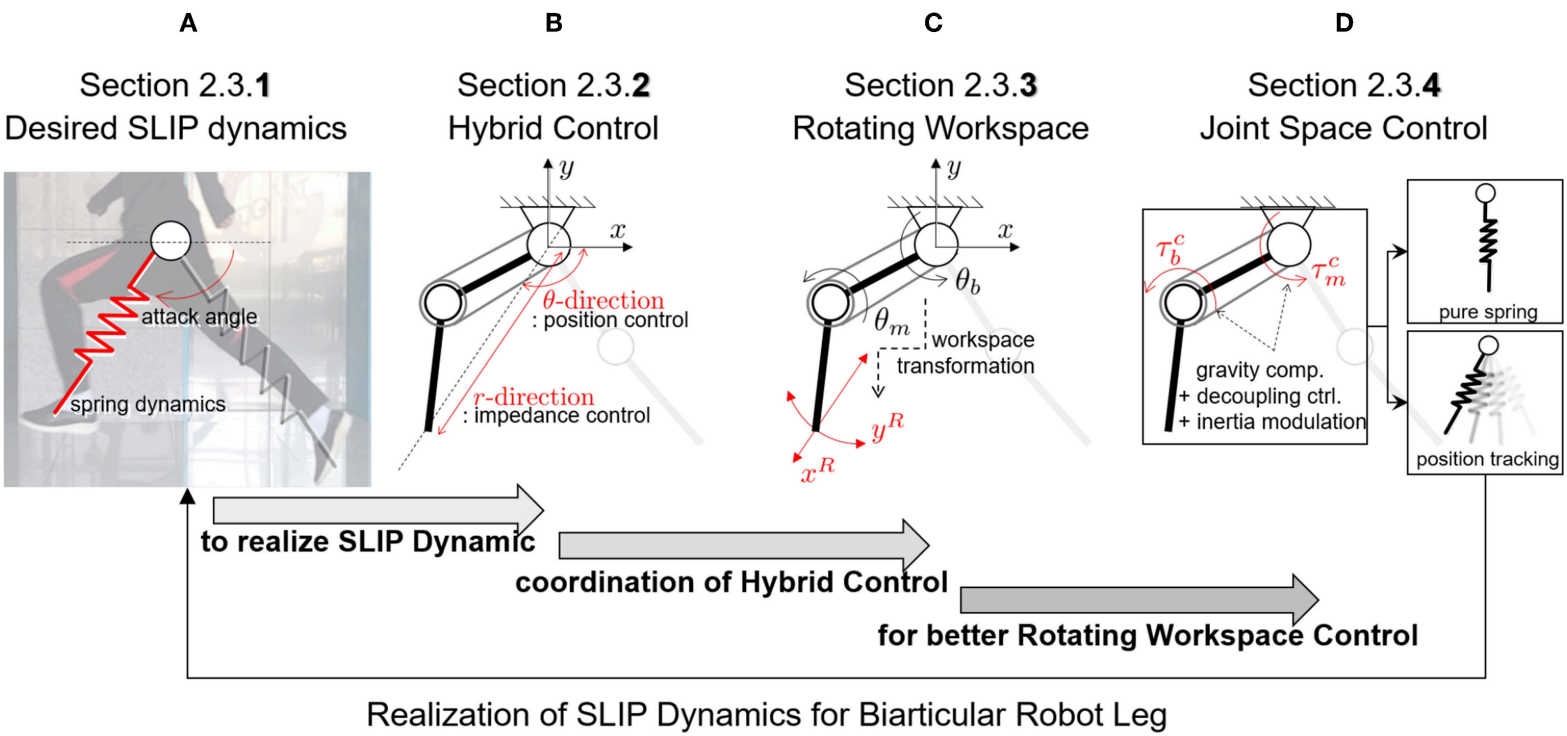
Organization and classification of contents in section 2.3. **(A)** Desired SLIP dynamics to realize human-like locomotion. **(B)** Hybrid Control strategy to achieve SLIP dynamics. **(C)** Rotating Workspace coordination for Hybrid Control. **(D)** Joint space control for better control performance.

This section introduces the control algorithm to realize human locomotive characteristics by using SLIP dynamics. As shown in Figure 11, the section includes four subsections as follows:

- **Section 2.3.1:** The control approach and its required technique are obtained to achieve the SLIP dynamics, which describes bio-inspired locomotion such as a running human.
- **Section 2.3.2:** The hybrid control methodology is proposed for the realization of SLIP dynamics, and the SLIP-oriented coordination of the biarticular robot leg, which is called Rotating Workspace, is defined concerning the hybrid controller implementation.
- **Section 2.3.3:** The kinematics, statics and dynamics of the biarticular robot leg are analyzed in the Rotating Workspace, and the reasons and problems that require the joint-space sub-control techniques for better hybrid control performance are explained.
- **Section 2.3.4:** The joint-space controllers are designed to improve the performance of hybrid control for pure SLIP behavior of the developed biarticular robot leg.

#### 2.3.1. Introduction of SLIP Model Based on Characteristic of Human Locomotion

Due to the recent improvements in the anthropometric techniques, human motions can be measured and analyzed in a theoretical way more than ever. One of the most simplified and well-formulated approaches to deriving the dynamic model of human walking and running focuses on the spring-like interaction of the leg with the ground during the stance phase and the free motion of the leg during the swing phase. The model that is developed to describe this feature is the Spring Loaded Inverted Pendulum model (Blickhan, 1989). Since the SLIP model simplifies the description and analysis of human locomotion, it is becoming a powerful template to realize human-like locomotion using various types of robots.

As described in Figure 11A, the main requirements to realize stable and periodic running of a robot are the realization of spring dynamics and attack angle control. These correspond to the SLIP model-based locomotion as 2-fold: a pure mass-spring behavior during the ground interaction or stance phase is rendered, and the correct attack angle with regards to the momentum of the mass at the landing moment or incidence angle of the leg is required.

One successful engineering approach to achieve the SLIP model-based running of a robot is a combination of a linear actuator for the interaction and a rotary actuator for the leg swing motion (Raibert, 1986). In this approach, the robot can realize SLIP model-based running even if the configuration of the robot is far from a human-like design. The robot is not developed to mimic the human muscular-skeletal configuration but to realize SLIP dynamics.

Articulated robot legs, such as the proposed robot leg, are realized by using the scientific approach to meet human-like locomotion specification. However, the conventional approach suffers from a technical bottleneck since the operational coordinate system of articulated robots differs from the operational space of SLIP dynamics, even though the configuration of the robot leg is derived from a bio-inspired design. Therefore, it is challenging to implement SLIP dynamics of the articulated robot directly.

**Figure 12:**
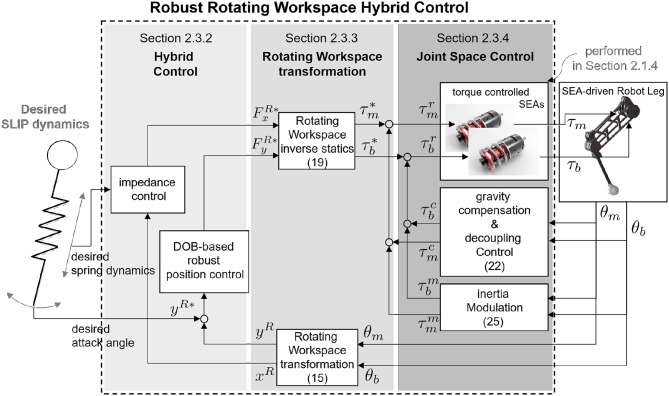
Overall control structure of the Robust RWHC for biarticular robot.

The novel point of this paper is the transformation of the operational coordinate to develop a complementary coordinate for SLIP motion. In other words, the operational coordinate system of the articulated leg has to be transformed to include the interactive operational and swing operational axes for the SLIP dynamics. In this perspective, the new Hybrid Control (HC) described in Figure 11B is designed in the Rotating Workspace (RW) described in Figure 11C to match the articulated robot with the operational space of SLIP dynamics. The overall control structure to realize SLIP dynamics is described in Figure 12. The proposed robust Rotating Workspace Hybrid Control (RWHC) algorithm consists of three layers, which are HC, Rotating Workspace transformation and joint-level controllers, and is shown as a dashed square in Figure 12. In the following sections, the details of these control parts are designed and analyzed.

#### 2.3.2. Proposed Approach to Realize SLIP Dynamics: Hybrid Control in Rotating Workspace

This subsection introduces the HC method, which is a control method to realize SLIP dynamics. HC refers to the simultaneous control of θ- and *r*-directions as shown in Figure 11B. These directions are the operation directions in RW and are required for realizing attack angle control and impedance control of the spring dynamics of SLIP in Figure 11A. As shown in Figure 11B, both the position controller with respect to *θ*-direction for the attack angle control and impedance control with respect to *r*-direction for the spring dynamics are implemented to accomplish HC in RW.

A robust position controller is chosen to overcome the influences of inherent coupling dynamics of the robot leg and external disturbances and to achieve precise attack angle control. For this requirement, the DOB-based two-DoF controller is adopted once more, but it uses a different dynamic model with the case of SEA torque control in section 2.1.4. The basic concept of this control approach has been proposed in Oh and Kong (2014).

The nominal inverse model of DOB is obtained based on a dynamic relationship between the *θ*-directional motion and the *θ*-directional force of the robot leg in the RW. In the viewpoint of the implementation, the statics and kinematics are also obtained to transform from the measured joint angles *θ*_*m*_ and *θ*_*b*_ to *θ*-directional motion and to generate the appropriate torques through the joint actuators. The coordinate transformation (kinematics) is required to project the reference position from the attack angle to the joint position.

On the other hand, the realization of the spring dynamics requires impedance control with respect to *r*-direction in the RW. The impedance control method enables rendering of desired dynamic behavior between the plant and the environment, e.g., the interaction of a second-order spring-mass system with the ground. There are two typical ways to render the mechanical impedance of the robot: position-based admittance control and force-based impedance control. The former needs a position-controlled robot system and end-effector force sensor, and the latter requires a force-controlled actuator system and joint position sensors. Since the proposed robot leg does not have an end-effector force sensor but includes force-controlled SEAs with measurable joint positions, the force-based impedance control is utilized to realize spring dynamics of the SLIP model with respect to *r*-direction in the RW. Moreover, in this the force-based impedance control, the inherent coupled dynamic behavior also appears on the closed-loop system in the RW. Therefore, the dynamics of the robot leg in the RW is analyzed for the proper rendering of the desired spring dynamics.

The *r*-directional impedance control law consists of the *r*-directional position displacement as a feedback signal and the reference impedance model. The *r*-direction position displacement can be calculated by using the kinematic relationship between the biarticular joint space angles and the *r*-directional motion, and the reference impedance model is defined based on the desired spring model of SLIP dynamics. Furthermore, the *r*-direction position displacement is multiplied by the reference impedance model to calculate the required r-directional force. The statics of the robot leg is required to generate the appropriate torques in the joint-space for rendering the reference impedance.

In summary, for the implementation of the proposed RWHC in the low-level joint control, workspace coordination—including the derivations of kinematic and statics—is required. Furthermore, the dynamics of the proposed robot leg in the RW is necessary for realization of the DOB-based position control and analysis of the behavior of the impedance-controlled system.

#### 2.3.3. Analysis of the Developed Robot Leg in Rotating Workspace for Hybrid Control

A novel operational space that is suitable for the coordination of the robot leg and the realization of spring dynamics and control of attack angle is needed to implement SLIP dynamics. The coordination that can perfectly match the biarticular joint space and the demands of SLIP dynamics is RW transformation, unlike the conventional workspace dynamics and its control which are designed in the Cartesian coordinate system.

In this section, the Jacobian, the statics, kinematics and the dynamics are defined in the RW, which conforms with the implementation of SLIP dynamics of the biarticular robot leg as follows:

- **Kinematics:** a kinematic relationship between the measured joint angles (*θ*_*m*_ and *θ*_*b*_) in the biarticular coordinate system and the inputs (*θ*- and *r*-directional motions) of HCler is obtained by using the positions in the reference coordinate system (and is utilized for RW control and Jacobian derivation).
- **Jacobian:**the coordinate relationship between the joint angular velocities (${\dot{\theta }}_{m}$ and ${\dot{\theta }}_{b}$) and end effector velocities (*ẋ*^*R*^ and *ẏ*^*R*^) is derived from a differentiation of kinematics (and is employed for RW dynamics derivation and statics derivation).
- **Statics:** force transmission from the joint actuator torques (*τ*_*m*_ and *τ*_*b*_) to end effector forces ($F_{x}^{R}$ and $F_{y}^{R}$) is derived from the Jacobian (and is employed for RW control and dynamics derivation).
- **RW dynamics:** The dynamic relationship between the biarticular joint torques and RW motion is obtained by utilizing Jacobian, statics and joint space dynamics in Equation (11) (and is utilized for analysis of the dynamic behavior and performance improvement of Rotating Workspace HC).

The RW is first introduced by Oh and Kong (2014). The paper provides the whole procedure for obtaining the transformation mathematics in detail and briefly shows the procedure for better understanding the controller design based on a RW formulation.

The kinematics between RW and biarticular joint-space is represented by a coordinate system that is rotated by the angle θ as shown in Figure 11B. The angle θ is the relative angle between the reference coordinate system and the robot endpoint position *r*, which are given by

(15) $$\begin{array}{l} r=\sqrt{P_{x}^{2}+P_{y}^{2}}=2l\cos\frac{1}{2}\left(\theta_{b}-\theta_{m}\right),\\ \theta =\tan^{-1}\frac{P_{y}}{P_{x}}=\frac{1}{2}\left(\theta_{b}+\theta_{m}\right) \end{array}$$

The physical representations of the coordinates in the RW can be exactly matched with the SLIP motion. The *r*-directional movement of the robot end effector corresponds to ground interactive motion and *θ*-directional movement corresponds to leg swing motion, i.e., determination of the attack angle of the SLIP dynamics.

The Jacobian is obtained by considering the end effector velocities in the RW, which is given by

(16) $$\begin{array}{l} {\dot{x}}^{R}=\dot{r} \end{array}$$

(17) $$\begin{array}{l} {\dot{y}}^{R}=r{\dot{\theta }}_{r} \end{array}$$

where *ẋ*^*R*^ denotes *r*-directional velocity and *ẏ*^*R*^ represents *θ*-directional velocity as shown in Figure 11C. The velocities can be described also by multiplication of the rotation matrix and biarticular Jacobian matrix ***J***_*b*_, and this yields the RW Jacobian ***J***_*R*_ as follows:

(18) $$\begin{array}{l} \left[\begin{array}{c} {\dot{x}}^{R}\\ {\dot{y}}^{R} \end{array}\right]=\left[\begin{array}{cc} \cos\theta & \sin\theta \\ -\sin\theta & \cos\theta \end{array}\right]J_{b}\left[\begin{array}{c} {\dot{\theta }}_{m}\\ {\dot{\theta }}_{b} \end{array}\right]\\ \;=l\left[\begin{array}{cc} \sin\frac{\theta_{b.r}}{2} & -\sin\frac{\theta_{b.r}}{2}\\ \cos\frac{\theta_{b.r}}{2} & \cos\frac{\theta_{b.r}}{2} \end{array}\right]\left[\begin{array}{c} {\dot{\theta }}_{m}\\ {\dot{\theta }}_{b} \end{array}\right]=J_{R}\left[\begin{array}{c} {\dot{\theta }}_{m}\\ {\dot{\theta }}_{b} \end{array}\right] \end{array}$$

By using the obtained RW Jacobian ***J***_*R*_, the static force relationship between the biarticular joint space torques and the RW forces can be described as follows:

(19) $$\begin{array}{l} \left[\begin{array}{c} \tau_{m}\\ \tau_{b} \end{array}\right]=J_{R}^{T}\left[\begin{array}{c} F_{x}^{R}\\ F_{y}^{R} \end{array}\right]=l\left[\begin{array}{cc} \sin\frac{\theta_{b.r}}{2} & \cos\frac{\theta_{b.r}}{2}\\ -\sin\frac{\theta_{b.r}}{2} & \cos\frac{\theta_{b.r}}{2} \end{array}\right]\left[\begin{array}{c} F_{x}^{R}\\ F_{y}^{R} \end{array}\right] \end{array}$$

where $F_{x}^{R}$ and $F_{y}^{R}$ indicate *r*-directional and *θ*-directional forces. The derived kinematics in Equation (15) and statics in Equation (19) enable the RW coordinate transformation in the middle layer of Figure 12 to realize RWHC. Using these, the leg dynamics in the RW is also derived for the analysis of the coupling behavior with inherent dynamic characteristics.

In order to obtain the dynamics of the robot leg in the RW, the coordinate description of accelerations *ẍ*^*R*^ and *ý*^*R*^ is obtained. The acceleration relationship of two operational spaces can be derived by time differentiation and rearrangement (from ***J***_*R*_ to $J_{R}^{-1}$) of Equation (18) as follows.

(20) $$\begin{array}{l} \left[\begin{array}{c} {\ddot{\theta }}_{m}\\ {\ddot{\theta }}_{b} \end{array}\right]=J_{R}^{-1}\left[\begin{array}{c} {\ddot{x}}^{R}\\ {\ddot{y}}^{R} \end{array}\right]-J_{R}^{-1}{\dot{J}}_{R}\left[\begin{array}{c} {\dot{\theta }}_{m}\\ {\dot{\theta }}_{b} \end{array}\right] \end{array}$$

where ${\dot{J}}_{R}$ denotes a time differentiation of the RW Jacobian. RW dynamics of biarticular actuator mechanism can be derived by using the inverse of Equation (19) and the Equation (20) as follows:

(21) $$\begin{array}{l} J_{R}^{T}\left[\begin{array}{c} F_{x}^{R}\\ F_{y}^{R} \end{array}\right]=\left[\begin{array}{c} \tau_{m}\\ \tau_{b} \end{array}\right]=\left[\begin{array}{cc} I_{m} & I_{c}\left(\theta_{b.r}\right)\\ I_{c}\left(\theta_{b.r}\right) & I_{b} \end{array}\right]\left[\begin{array}{c} {\ddot{\theta }}_{m}\\ {\ddot{\theta }}_{b} \end{array}\right]\\ \;+\left[\begin{array}{c} \tau_{m.c}\left(\theta_{b.r},{\dot{\theta }}_{b}\right)+\tau_{m.g}\left(\theta_{m}\right)\\ \tau_{b.c}\left(\theta_{b.r},{\dot{\theta }}_{m}\right)+\tau_{b.g}\left(\theta_{b}\right) \end{array}\right],\\ \;[\begin{array}{c} F_{x}^{R}\\ F_{y}^{R} \end{array}]={\left(J_{R}^{T}\right)}^{-1}\left[\begin{array}{cc} I_{m} & I_{c}\left(\theta_{b.r}\right)\\ I_{c}\left(\theta_{b.r}\right) & I_{b} \end{array}\right]J_{R}^{-1}\left(\left[\begin{array}{c} {\ddot{x}}^{R}\\ {\ddot{y}}^{R} \end{array}\right]-{\dot{J}}_{R}\left[\begin{array}{c} {\dot{\theta }}_{m}\\ {\dot{\theta }}_{b} \end{array}\right]\right)\\ \;+{\left(J_{R}^{T}\right)}^{-1}\left[\begin{array}{c} \tau_{m.c}\left(\theta_{b.r},{\dot{\theta }}_{b}\right)+\tau_{m.g}\left(\theta_{m}\right)\\ \tau_{b.c}\left(\theta_{b.r},{\dot{\theta }}_{m}\right)+\tau_{b.g}\left(\theta_{b}\right) \end{array}\right] \end{array}$$

**Figure 13:**
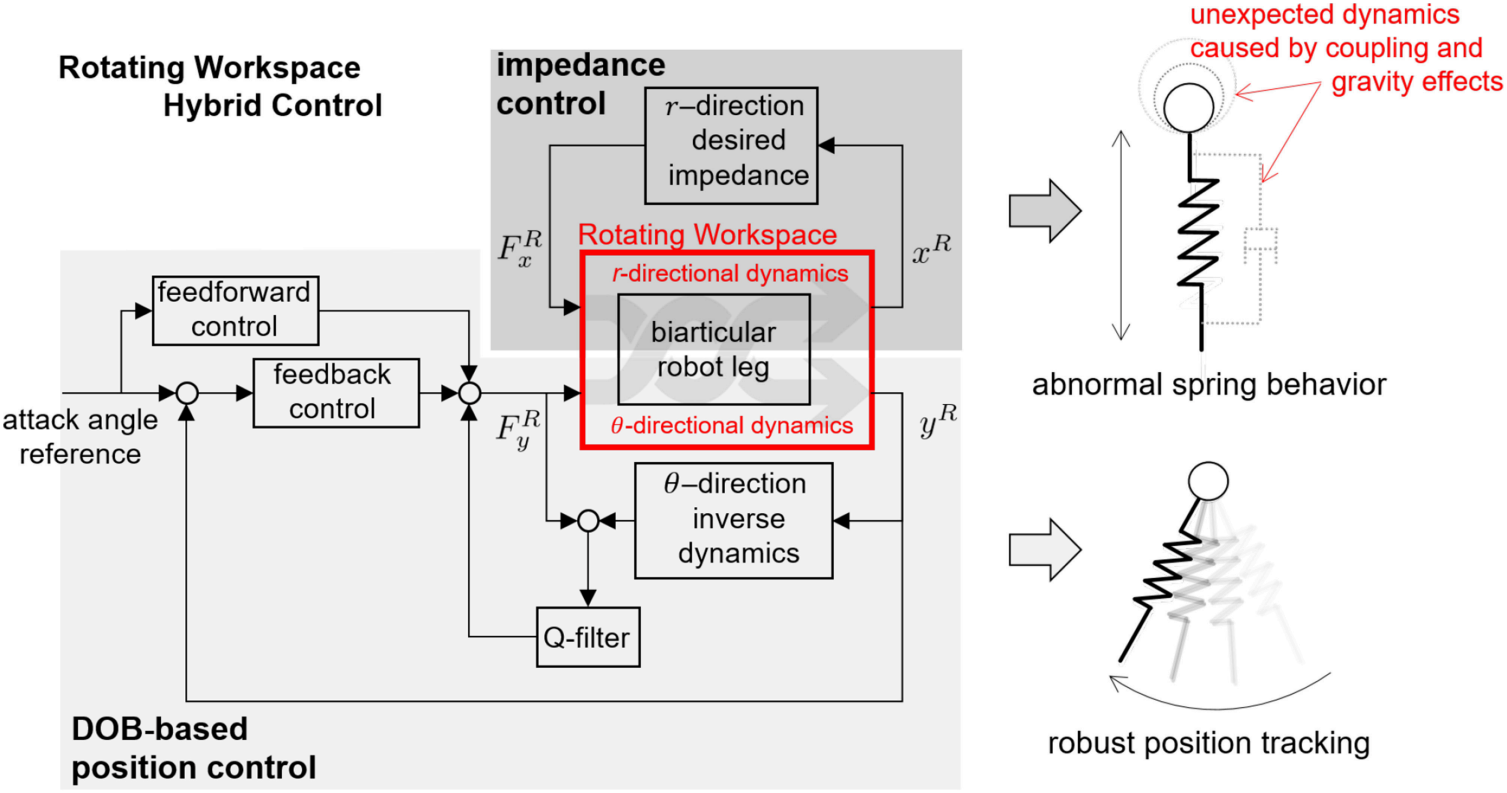
HC in the RW for biarticular robot leg.

Based on Equation (21), it can be concluded that the RW coordinate corresponds kinematically to SLIP motion. However, the dynamics is complicatedly coupled in the RW coordinate. Because of these dynamic characteristics (dynamics coupling and gravity effect), the motions in the RW, *x*^*R*^ and *y*^*R*^, which are to be controlled, adversely affect each other. Therefore, the performance of HC is deteriorated as shown in Figure 13. The next section shows how to remove the dynamic coupling effect and improve its performance effectively.

#### 2.3.4. Joint Space Dynamic Decoupling to Improve Performance of Robust Rotating Workspace HC

As described above, the attack angle control for the SLIP dynamics can be implemented using the high gain feedback without consideration of dynamics behavior in the RW. However, for the realization of the spring behavior of SLIP dynamics, it is not straightforward to render the desired dynamic behavior without considering the influence of mechanical characteristics such as inertia coupling and gravity effect. In this section, systematic dynamic decoupling methods in the joint space are designed to enhance the performance of the proposed RWHC based on the structural advantage of the biarticular robot leg.

A study on dynamics modification such as dynamic decoupling for a general multi-link robot has been first proposed in Khatib (1987). Khatib has developed the ‘Operational Space Nonlinear Dynamic Decoupling’ method to directly modify the robot's dynamic characteristics based on an identification of the coupling and gravity terms appearing in the workspace without using the structural features. The method requires correct identifications of complicated inertia coupling, Coriolis and gravity terms; hence, it is not effective to directly apply Khatib's approach to the biarticular robot leg.

In order to decouple the dynamics of the leg effectively, a systematic approach is required based on a structural advantage of the biarticular robot leg. In section 2.2.2, the structural advantage was found to be that the robot legs on the biarticular coordinate system are configured to be identified easily. Therefore, the proposed method utilizes joint level identification and decoupling in the biarticular coordinate to simplify the complex dynamics in Equation (21) (detailed in section 2.3.4.1). Furthermore, inertia control is applied to the simplified joint space dynamics to achieve better HC performance in the RW (detailed in section 2.3.4.2).

##### 2.3.4.1. Compensation of Inherent Dynamics - Inertia Decoupling and Gravity Compensation

As mentioned before, because of the torque control, measurement capabilities of the SEA and biarticular actuator mechanism, the identification and compensation of the gravity, Coriolis and inertia torque in the joint space are simpler than in RW. Hence, the compensation of inherent dynamic coupling is performed in the joint space.

The controller for inertial and gravity compensation is designed based on the joint-space dynamics derived in Equations (12)–(14). Based on these equations, required compensation torques $\tau_{m}^{c}$ and $\tau_{b}^{c}$ are obtained as follows:

(22) $$\begin{array}{l} \tau_{m}^{c}=C_{I}\cos\theta_{b.r}{\ddot{\theta }}_{b}-C_{I}\sin\theta_{b.r}{\dot{\theta }}_{b}^{2}+G_{m}\cos\theta_{m}\\ \tau_{b}^{c}=C_{I}\cos\theta_{b.r}{\ddot{\theta }}_{m}+C_{I}\sin\theta_{b.r}{\dot{\theta }}_{m}^{2}+G_{b}\cos\theta_{b} \end{array}$$

*θ*_*m*_ and *θ*_*b*_ are measured by position sensors of each joint SEA and *θ*_*b*.*r*_ is calculated by Equation (4). ${\dot{\theta }}_{m}$, ${\dot{\theta }}_{b}$, ${\ddot{\theta }}_{m}$ and ${\ddot{\theta }}_{b}$ can be calculated by numerical differentiation of *θ*_*m*_ and *θ*_*b*_. Three coefficients, *G*_*m*_, *G*_*b*_ and *C*_*I*_, are required to implement this compensation; however, the gravity term of biarticular-side joint *G*_*b*_ includes the common coupling coefficient *C*_*I*_ as derived in Equation (14). Therefore, simple identification of *G*_*m*_ and *G*_*b*_ enable this compensation. Note that the identification method for *G*_*m*_ and *G*_*b*_ is described in section 3.3.

This compensation results in a fully decoupled joint-space dynamics in the biarticular coordinate system as follows:

(23) $$\begin{array}{l} \left[\begin{array}{c} \tau_{m}-\tau_{m}^{c}\\ \tau_{b}-\tau_{b}^{c} \end{array}\right]=\left[\begin{array}{cc} I_{m} & 0\\ 0 & I_{b} \end{array}\right]\left[\begin{array}{c} {\ddot{\theta }}_{m}\\ {\ddot{\theta }}_{b} \end{array}\right]={\text{I}}_{bi}\left[\begin{array}{c} {\ddot{\theta }}_{m}\\ {\ddot{\theta }}_{b} \end{array}\right], \end{array}$$

where **I**_*bi*_ is the diagonal inertia matrix. $\tau_{m}-\tau_{m}^{c}$ and $\tau_{b}-\tau_{b}^{c}$ are the new torque references for the closed loop system with the proposed compensation in Equation (22). Because of the compensation, the dynamics of the joint-space are decoupled and simplified.

In order to analyze the effect of the compensation in RW, the modified dynamics is obtained in a similar way as in Equation (21).

(24) $$\begin{array}{l} \left[\begin{array}{c} F_{x}^{R*}\\ F_{y}^{R*} \end{array}\right]={\left(J_{R}^{T}\right)}^{-1}{\text{I}}_{bi}J_{R}^{-1}\left(\left[\begin{array}{c} {\ddot{x}}^{R}\\ {\ddot{y}}^{R} \end{array}\right]-{\dot{J}}_{R}\left[\begin{array}{c} {\dot{\theta }}_{m}\\ {\dot{\theta }}_{b} \end{array}\right]\right)\\ \;=\left[\begin{array}{cc} \frac{I_{m}}{l-l\cos\theta_{b.r}} & 0\\ 0 & \frac{I_{b}}{l+l\cos\theta_{b.r}} \end{array}\right]\left(\left[\begin{array}{c} {\ddot{x}}^{R}\\ {\ddot{y}}^{R} \end{array}\right]-{\dot{J}}_{R}\left[\begin{array}{c} {\dot{\theta }}_{m}\\ {\dot{\theta }}_{b} \end{array}\right]\right) \end{array}$$

where $F_{x}^{R*}$ and $F_{y}^{R*}$ are the new force reference regarding the RW coordinates with the proposed compensation in Equation (22). ${\left(J_{R}^{T}\right)}^{-1}{\text{I}}_{bi}J_{R}^{-1}$ indicates operational space inertia (Khatib, 1987).

Even though the inertial compensation is achieved in Equation (24), the diagonal components of the inertia matrix vary with *θ*_*b*.*r*_. These variations deteriorate the performance of RWHC. In particular, the variation of the first inertia term $\frac{I_{m}}{l-l\cos\theta_{b.r}}$, which is the projected inertia with respect to *r*-direction, affects the realization of spring behavior of SLIP dynamics. In order to solve this phenomenon, the first inertia term should be controlled as a fixed inertia by adding a separate inertia control.

##### 2.3.4.2. Inertia Control for Robust Rotating Workspace Hybrid Control

In this subsection, the additional inertia control is designed to fix *r*-directional inertia for better performance of RWHC.

In order to fix the first term $\frac{I_{m}}{l-l\cos\theta_{b.r}}$ in an operational space inertia matrix, *I*_*m*_ should be modulated to include the term *l* − *l*cos *θ*_*b*.*r*_. Therefore, the goal of inertial control can be defined as modulating the joint-space inertia *I*_*m*_ as $M_{d}^{R}\left(l-l\cos\theta_{b.r}\right)$, where $M_{d}^{R}$ is the desired inertia (mass) of the SLIP model.

The result of the compensated dynamic system in the biarticular actuator coordinates is linear and is simple enough as shown in Equation (23). Hence, the inertia control can be easily implemented in the biarticular joint-space. The required joint torques $\tau_{m}^{m}$ and $\tau_{b}^{m}$ to modulate the inertia *I*_*m*_ as $M_{d}^{R}\left(l-l\cos\theta_{b.r}\right)$ are given by

(25) $$\begin{array}{l} \left[\begin{array}{c} \tau_{m}^{m}\\ \tau_{b}^{m} \end{array}\right]=\left[\begin{array}{cc} M_{d}^{R}\left(l-l\cos\theta_{b.r}\right)-I_{m} & 0\\ 0 & M_{d}^{R}\left(l-l\cos\theta_{b.r}\right)-I_{b} \end{array}\right]\left[\begin{array}{c} {\ddot{\theta }}_{m}\\ {\ddot{\theta }}_{b} \end{array}\right], \end{array}$$

Note that the second term of inertia control can be designed in another way; however, this paper chooses $M_{d}^{R}\left(l-l\cos\theta_{b.r}\right)-I_{b}$ for simplicity.

By applying the inertia control to fix the *r*-directional inertia, the dynamics in Equation (24) can be reformulated in the RW by utilizing the RW Jacobian and statics in Equations (18) and (19) as follows:

(26) $$\begin{array}{l} \left[\begin{array}{c} F_{x}^{R*}\\ F_{y}^{R*} \end{array}\right]=\left[\begin{array}{cc} M_{d}^{R} & 0\\ 0 & M_{d}^{R}\tan^{2}\frac{\theta_{b.r}}{2} \end{array}\right]\left[\begin{array}{c} {\ddot{x}}^{R}\\ {\ddot{y}}^{R} \end{array}\right]\\ \;+\;\frac{1}{2}\left[\begin{array}{cc} M_{d}^{R}\left(\cos\theta_{b.r}-1\right){\dot{\theta }}_{b.r} & 0\\ 0 & M_{d}^{R}\left(1-\cos\theta_{b.r}\right){\dot{\theta }}_{b.r} \end{array}\right]\left[\begin{array}{c} {\dot{x}}^{R}\\ {\dot{y}}^{R} \end{array}\right] \end{array}$$

Even though the closed-loop dynamic behavior in Equation (26) with respect to *r*-direction contains the damping-like term $\frac{1}{2}M_{d}^{R}\left(\cos\theta_{b.r}-1\right){\dot{\theta }}_{b.r}$, the inertia is fixed and decoupled as desired inertia $M_{d}^{R}$. For the perfection of spring behavior of SLIP model, the *r*-directional impedance model of HC can be redesigned to compensate the remaining damping-like effect as

(27) $$\begin{array}{l} F_{x}^{R*}=-K_{d}^{R}x^{R}-\frac{1}{2}M_{d}^{R}\left(\cos\theta_{b.r}-1\right){\dot{\theta }}_{b.r}{\dot{x}}^{R} \end{array}$$

where desired spring constant $K_{d}^{R}$ and mass $M_{d}^{R}$ are given by the desired SLIP model. This redesigned RWHC with joint level controls including decoupling control, gravity compensation and inertia modulation renders pure mass-spring behavior with respect to *r*-direction.

Interestingly, the *θ*-directional inertia in Equation (26) is also simplified as $M_{d}^{R}\tan^{2}\frac{\theta_{b.r}}{2}$. Considering that the leg posture is mainly placed in a common position, i.e., $\theta_{b.r}\approx \frac{\pi }{2}$, the term $\tan^{2}\frac{\theta_{b.r}}{2}$ in the inertia can be regarded as 1. Therefore, the nominal inverse model in the DOB in Figure 13 can be defined as $M_{d}^{R}s^{2}$. Even though the *r*-directional motion driven by impedance control of HC causes variation of *θ*-directional dynamic behavior (because of *θ*_*b*.*r*_), dynamics variation and unmodeled terms can be actively rejected as a disturbance by the ability of DOB. Therefore, the developed SEA-driven robot leg with the proposed RWHC, including two-axes controllers, realizes SLIP dynamics robustly.

**Figure 14:**
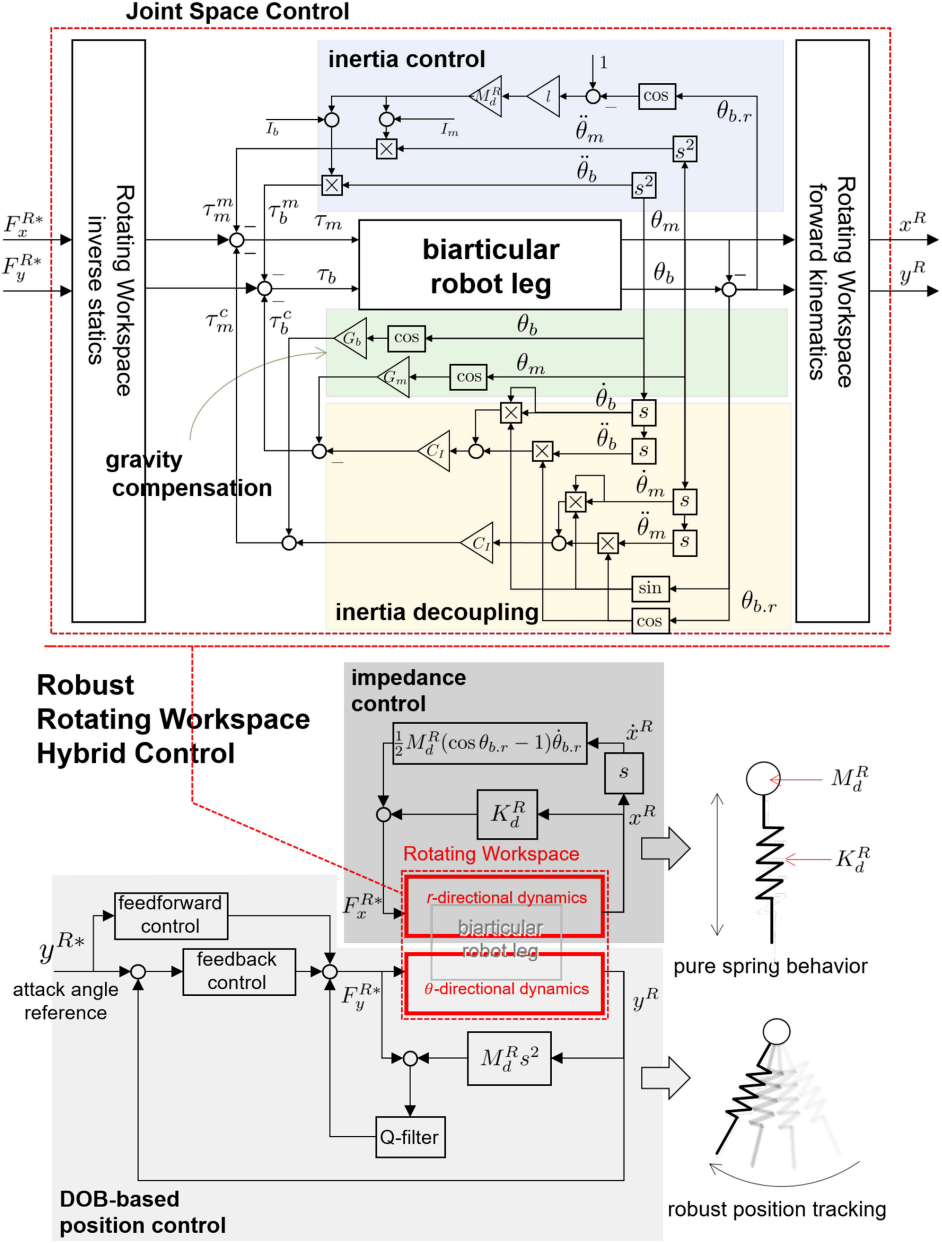
Robust HC in the RW for biarticular robot leg. The final form of the controller consists of inertia decoupling, gravity control and inertia control.

The overall structure of the Robust HC in the RW for biarticular robot leg is described in Figure 14.

## 3. Results

### 3.1. Experimental Realization of Robust Rotating Workspace Hybrid Control

Experiments are conducted using the developed SEA-driven robot leg to verify the following points:

1. **Hardware part:** the developed SEA-driven robot leg takes full advantage of SEA and the biarticular mechanism in terms of ease of parameter identification, force transmissibility and Rotating Workspace coordination.
2. **Software part:** Robust Rotating Workspace Hybrid Control consisting of the decoupling control, inertia modulation, impedance control and DOB-based position control is effectively applied to a robot leg.

The experiments are conducted by way of the following four steps to systematically verify the contribution points above.

**Section 3.3** System identification using motor position control of SEA
**Section 3.4** Verification of Statics using gravity compensation and Rotating Workspace force control of the robot leg
**Section 3.5** Verification of inertia decoupling control using decoupling control in the biarticular coordinate
**Section 3.6** Performance verification of Robust Rotating Workspace Hybrid Control investigating the effectiveness of inertia modulation in the biarticular coordinate, impedance control and position control in the Rotating Workspace

### 3.2. Experimental Setup

**Figure 15:**
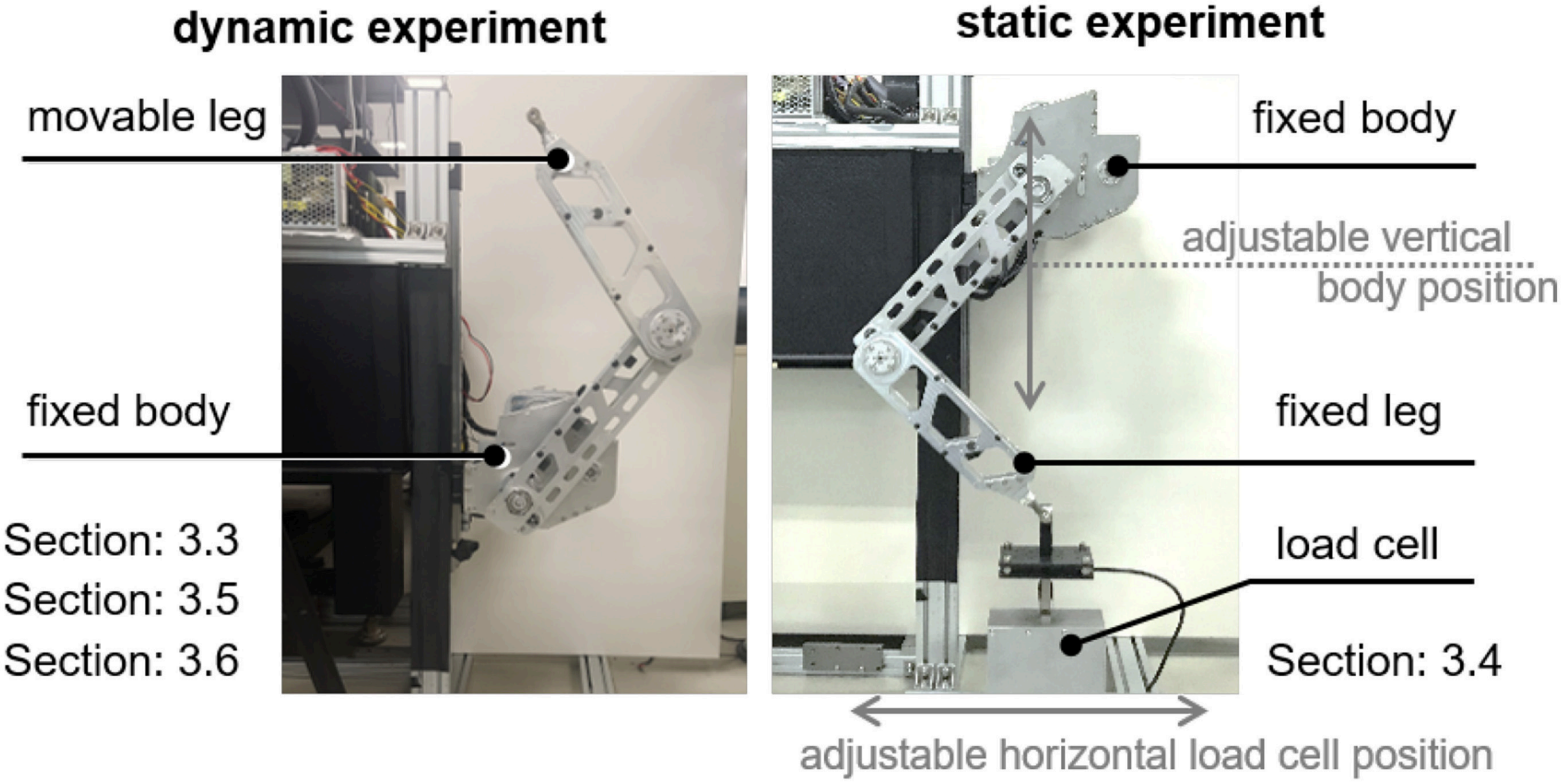
Experimental setups. Dynamic experiment: fixed body and freely moving leg. Static experiment: fixed body and leg with load cell.

The experiments for the verifications are set in two ways: the dynamic experiment and static experiment. The experimental setups are shown in Figure 15.

The setup for the dynamic experiment in the left-hand side of Figure 15 is utilized for the system identification and performance verification of proposed HC with joint level control. Then, the robot leg moves according to the control algorithms. Note that the system identification in section 3.3 uses this experimental setup for a variety of leg postures. However, the actual experimental behavior can be more properly described as ‘quasi-static’ than ‘dynamic’ (detailed experimental protocol is given in section 3.3).

The setup for the static experiment in the right-hand side of Figure 15 is utilized for verification of the force transmissibility from SEAs to the end effector through the biarticular mechanism and Rotating Workspace transformation. For this purpose, the endpoint of the leg is connected to a load cell through a universal joint, which distresses any unexpected kinematic constraint in the setup. A single axis (vertical direction) load cell is chosen to measure the vertical force exerted from the endpoint. The load cell (CAS, SBA-50L) used in this setup has a resolution that can measure up to 0.02 N of force with low-pass filtering (100 Hz). In this setup, the body position and the load cell position can be adjusted with respect to the vertical guide and the horizontal guide to change the leg posture.

### 3.3. System Identification of SEA-Driven Robot Leg With Biarticular Actuator Coordination

**Figure 16:**
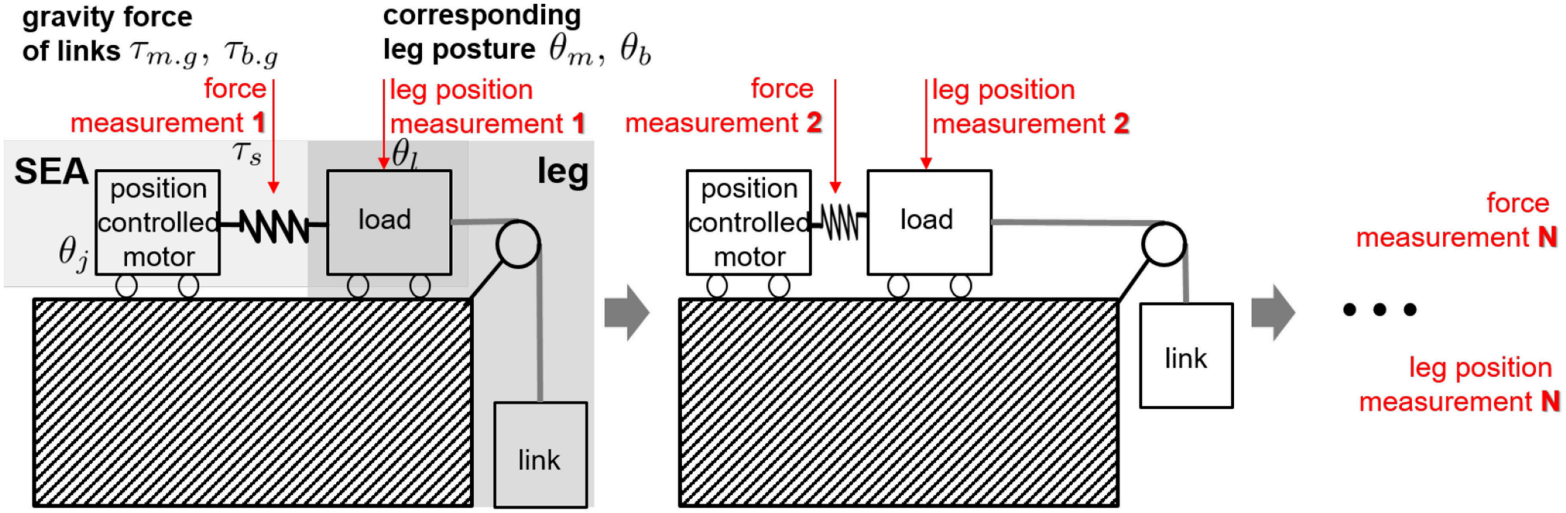
System identification procedure of the robot leg by utilizing the force, position measurement and position control capabilities of SEA.

**Figure 17:**
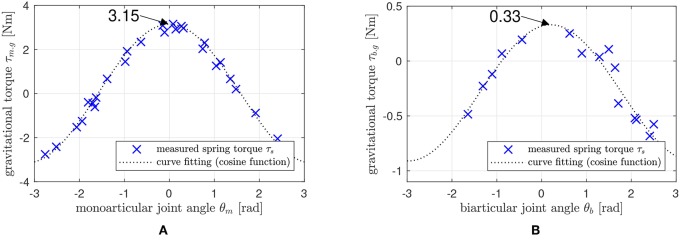
Gravity coefficient identification result. **(A)**: relationship between *θ*_*m*_ and *τ*_*m*.*g*_, and **(B)**: Relationship between *θ*_*b*_ and *τ*_*b*.*g*_.

As explained in section 2.2.2, the coefficients *G*_*m*_ and *G*_*b*_ of the gravity terms in (Equation 14) are characteristic parameters, which also include the inertia coupling coefficient *C*_*I*_. The identification of the gravity coefficient is a vital identification process for the whole system. Moreover, the SEA in the proposed robotic leg enables measurement of the interacting torques and gravitational torques by the link weights for the system identification. Taking advantage of these two points, the system identification of the robotic leg is proceeded as follows (in Figure 16); the motor-side angle (*θ*_*j*_ in Figure 7) is controlled to be constant, and the spring torque (*τ*_*s*_ in Figure 7) is measured after the system becomes steady state. Then, the load angle *θ*_*l*_ in Figure 7 is utilized as the mono and biarticular joint angle values of *θ*_*m*_ and *θ*_*b*_, and the measured spring torque *τ*_*s*_ is utilized as the mono and biarticular gravitational torques *τ*_*m*.*g*_ and *τ*_*b*.*g*_. This measurement process has been conducted with several different angles of *θ*_*j*_s, and Figure 17 shows the results of the measured torques *τ*_*s*_ and angles *θ*_*l*_. Two results are plotted as the measurement results using two SEAs: one for monoarticular joint and the other for the biarticular joint.

The plots show the relationship between *θ*_*l*_(= *θ*_*m*_, and = *θ*_*b*_) and *τ*_*s*_(= *τ*_*m*.*g*_, and = *τ*_*b*.*g*_) and exhibit a cosine function pattern, which corresponds to the derived relationship in Equation (14). By fitting the curves with appropriate scale selection, the gravity coefficients Gm and Gb can be identified. In this case, the coefficient of monoarticular joint gravity term *G*_*m*_ is identified as 3.15 Nm, and the biarticular joint coefficient $G_{b}=\frac{g}{l}C_{I}$ is identified as 0.33 Nm. Therefore, the coupling inertia term *C*_*I*_ is calculated as 0.01 $\frac{\text{N}}{{\text{m/s}}^{2}}$ based on the parameter values *g* = 9.81m/s^2^ and *l* = 0.3*m*.

### 3.4. Statics Verification of the Proposed Robot Leg With Biarticular Actuator Coordination

**Figure 18:**
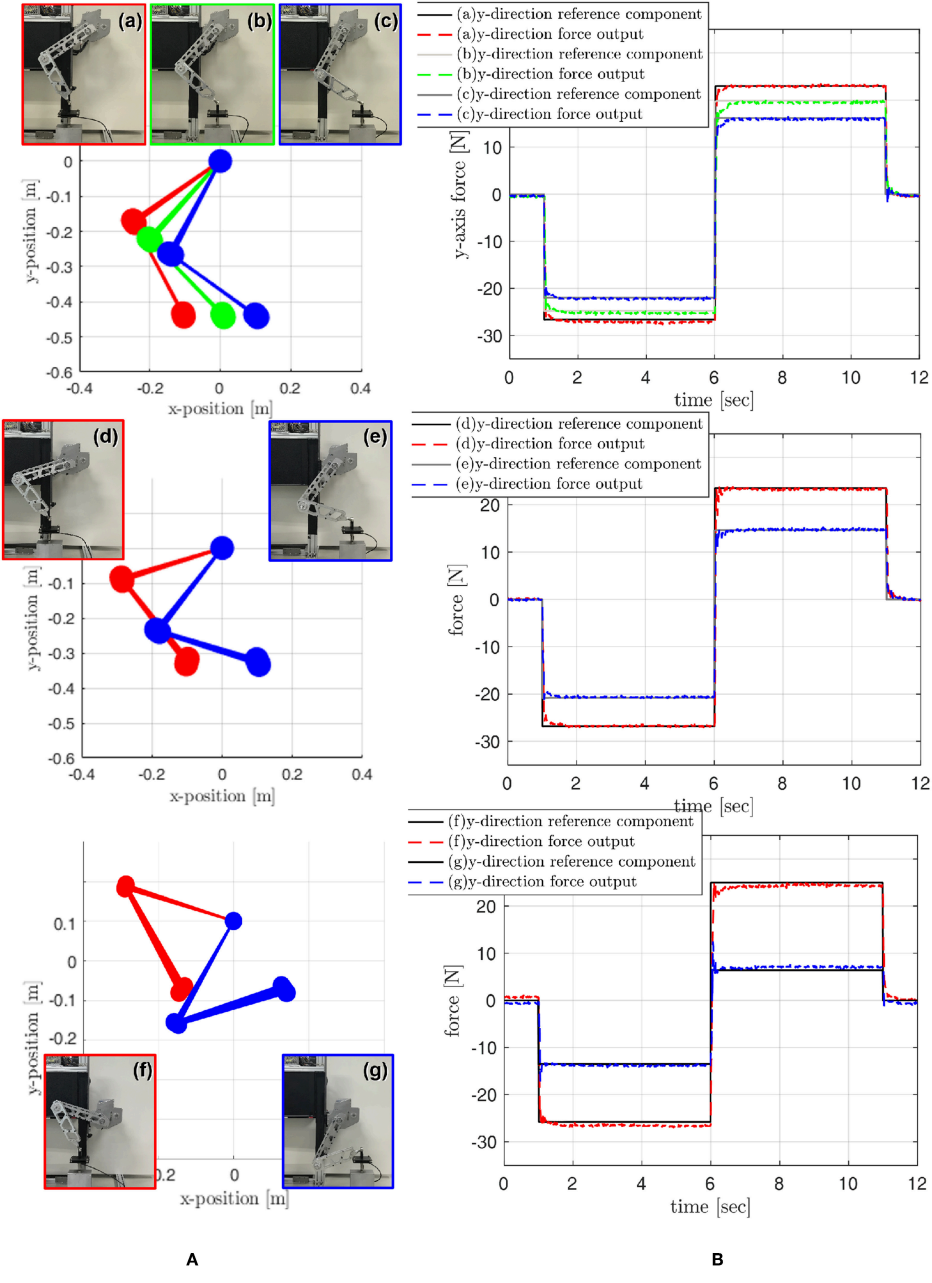
Verification of Static with various robot postures. **(A)** robot leg postures, and **(B)** end point force tracking performance.

The statics for the biarticular actuator torque coordinate and the force in the Rotating Workspace given in Equation (19) are verified through experiments. The joint torques *τ*_*m*_ and *τ*_*b*_ are provided by SEA torque control, and the end effector force is measured by the load cell. The relationship between the provided joint torques and the measured end effector force is compared to verify Equation (19). Various poses of the robot leg are set as shown in (a–g) of Figure 18. Notice that the gravitational force is compensated in the following experiments utilizing the identified Ĝ_*m*_ and Ĝ_*b*_.

Statics is verified through the force tracking at the end point where step-wise force references are given in the Rotating Workspace, and these references are converted to the required torque references for SEAs. The required torques are generated by SEA torque control, and the force generated at the end-point by these SEA torques are measured by the load cell and compared with the initial references.

Figure 18 shows the results of the force tracking and thus the statics verification. Various force references are given changing directions as shown in the right graphs of Figure 18. The black-solid lines in the right plots are the reference signals, and the dash-colored lines indicate the sensor measured forces.

The rise time, which measures the rate of the rise from 10 to 90% of the steady-state response, is calculated and averaged based on the measured data sets, and the result is 6.1 ms. The averaged root mean square error (RMSE) is also calculated, and the result is about 0.15 N. Note that the responses appear negligible steady-state errors in most cases; however, case (f) indicates about 1 N of steady state error, and this may be caused by misalignment between load cell and leg in the experimental setup with respect to critical posture of the leg.

The results imply that the force of the endpoint in the Rotating Workspace can be controlled using statics in Equation (19) and the high-performance torque control of SEAs. In particular, the results show that the torque control of the SEA can guarantee the stability and fast response time for the step-wise reference with a stiff environment.

### 3.5. Performance of Decoupling Control in the Biarticular Coordinate

Dynamics of the robotic leg with the biarticular actuator coordination can be decoupled as Equation (23) by applying decoupling control (Equation 22). The performance of this decoupling control is examined in this experiment.

The experimental setup and protocol are given as follows: a sinusoidal torque reference is provided to only one SEA to generate only one torque input *τ*_*m*_ or *τ*_*b*_, and the dynamic behavior of all joint angles *θ*_*m*_ and *θ*_*b*_ are examined with and without decoupling control (Equation 22).

A sinusoidal torque with an amplitude of 5 Nm and a frequency of 3 Hz is generated by a SEA of the specific joint (*τ*_*m*_ or *τ*_*b*_) as an excitation. In addition to this excitation torque, additional decoupling control torques designed as in Equation (22) are added to both SEAs, and all the joint angular velocities ${\dot{\theta }}_{m}$ and ${\dot{\theta }}_{b}$ are measured under this condition. After several seconds (1.5 s in the following experiments) of excitation, the decoupling control torques are turned off, and the change of the joint angular velocities are measured and compared with the behavior of the decoupling control.

**Figure 19:**
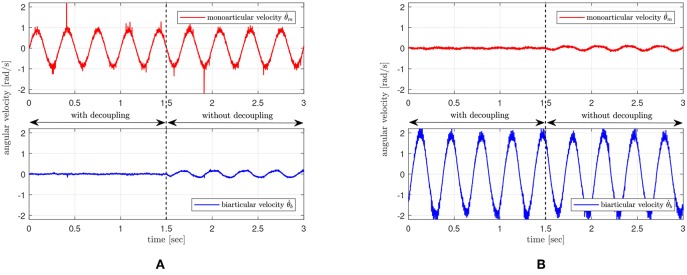
Performance verification of the proposed decoupling control. **(A)** excitation signal on monoarticular actuator, and **(B)** excitation signal on biarticular actuator.

Figure 19 shows the results of the decoupling control. The upper graphs are the angular velocities ${\dot{\theta }}_{m}$ of the monoarticular joint, and the bottom graph is the angular velocity ${\dot{\theta }}_{b}$ of the biarticular joint. The left result in Figure 19A is when the excitation signal is given to the monoarticular SEA *τ*_*m*_, and the right result in Figure 19B is when the excitation signal is given to *τ*_*b*_.

The comparison of the unexcited joint velocities before and after 1.5 s shows that unexcited joints in the both cases are not affected by the motion of the other joint. This result verifies that the proposed decoupling control can successfully decouple the effect from the other joint torque.

### 3.6. Robustness and Tracking Performance Verification of Rotating Workspace Hybrid Control for Realization of SLIP

The performance of the proposed Rotating Workspace Hybrid Control to realize SLIP template-based movement is verified through experiments. To assess the performance in detail, the following points are investigated.

1. Impedance control performance of the robot leg in the radial direction
2. High fidelity position tracking in the tangential direction
3. Robustness of radial directional impedance control during high accuracy tangential control

For this purpose, two types of experiments are designed.

Firstly, the leg is dragged and released in the radial direction to examine the controlled impedance against external perturbation. The results of dynamic behavior of the leg are compared with the theoretic solution that is analytically calculated based on the reference impedance model. The several types of reference impedance models implemented on the leg vary with the stiffness and damping values, which are 100, 150, and 250 N/m (low, mid and high stiffness with mid damping), and 5, 15, 25 Ns/m (low, mid, and high damping with mid stiffness). This varying stiffness and damping setting enables verification of the anti-inertia variation effect of the inner-loop inertia modulation control in the biarticular coordinate. The desired inertia $M_{d}^{R}$ in Rotating Workspace is set to 1.5 Kg, the length of the leg in the radial directional is set to 0.4 m, and the end point is dragged by a hand as far as 0.25 m.

**Figure 20:**
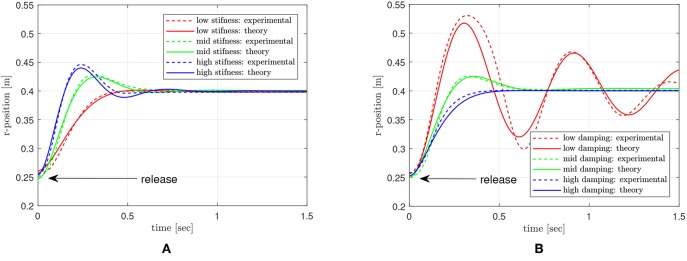
Dynamic response of the end point with different impedance setting. **(A)** various stiffness settings, and **(B)** various damping settings.

Figure 20 is the result of the drag-and-release test, which shows the behavior of the end point in the radial direction. The graph in Figure 20A shows the result with varying stiffness conditions, and the graph in Figure 20B shows the result with varying damping conditions. The red-, green- and blue-colored lines indicate low, mid and high stiffness/damping cases, respectively. The solid lines are the theoretical responses, and the dashed lines are the measured responses. Most of the experimental results match well with theoretical responses except the low damping case, which suffers from the nonlinear behavior after first oscillation. Even though the magnitude of the response is different from the theoretical model in this case, the natural frequencies are still well matched with the response of theoretical models.

Second, the dynamic response in the radial direction is investigated while the motion in the tangential direction is controlled. To examine the dynamic behavior of the end point, it is pushed in the radial direction, and the response in the radial direction against this pushing perturbation is measured and investigated. During this push and recovery process in the radial direction, the end point is position-controlled in the tangential direction. The reference for this motion is a sinusoidal pattern with 2 Hz frequency and 0.2 rad magnitude. The reference stiffness and damping for the radial direction impedance are set to 75 N/m and 10 Ns/m. In this experiment, the end point is pushed and recovered two times. In the first push-recovery process, the inertia modulation and the decoupling control are turned off, and it is turned on in the second push-recovery process.

**Figure 21:**
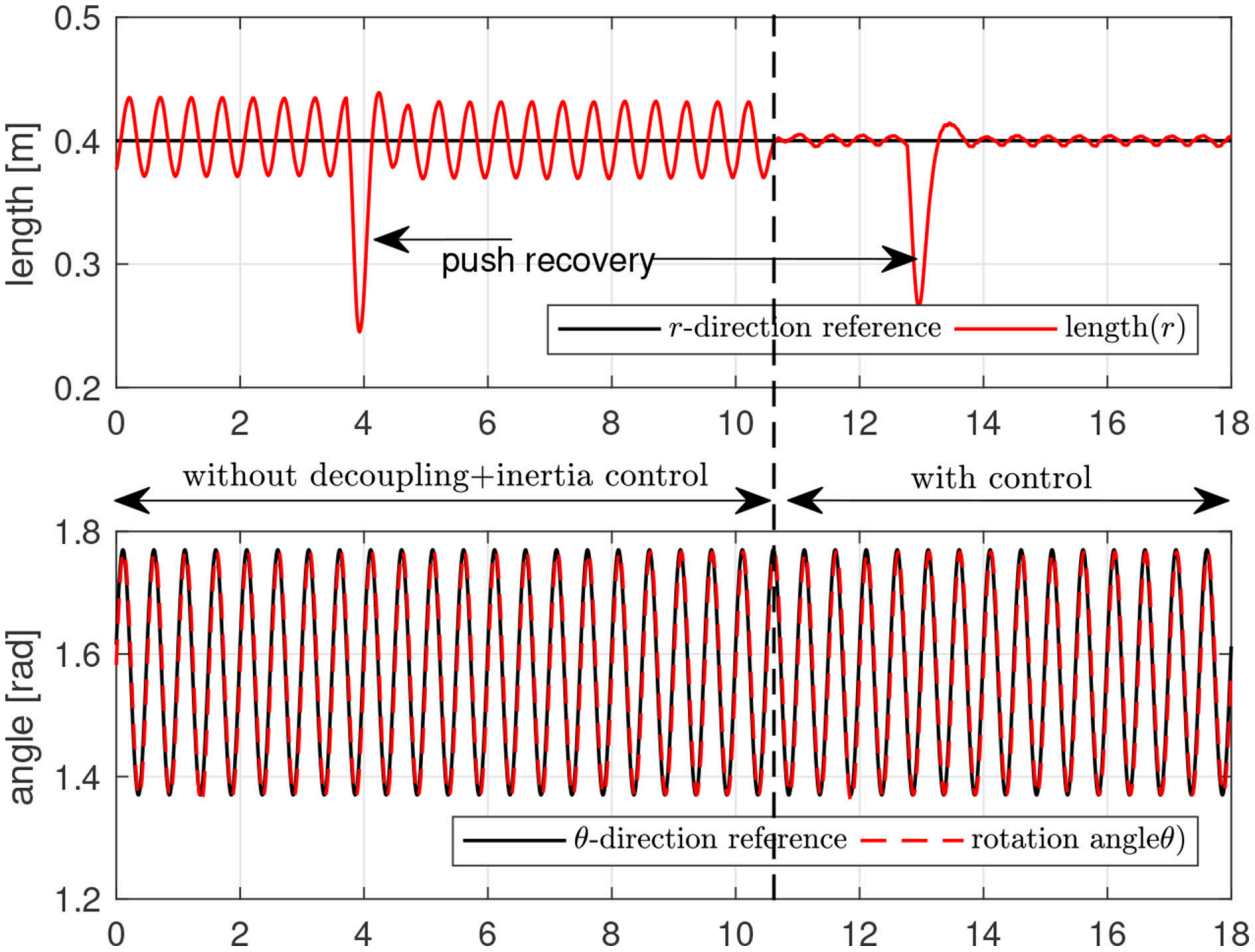
Robustness and tracking performance verification of Rotating Workspace Hybrid Control.

Figure 21 is the result of this experiment. The upper graph shows the position of the end point in the radial direction, while the bottom graph shows the position in the tangential direction. The end point is pushed at 4 s at first, and it is pushed again at 13 s. Even though the endpoint recovers after the first push, tt can be verified in that the radial motion suffers from the coupling by the tangential motion up to 10.4 s.

At 10.4 s, the decoupling control and inertia modulation control are switched on, and the coupling in the radial motion decreases dramatically. The radial motion exhibits the dynamic response designed by the radial impedance control against the second push. While the two pushing perturbations work in the radial direction, the tangential motion is not affected by the perturbation in the radial direction, which validates the robustness of the tangential position control.

## 4. Discussion and Conclusion

### 4.1. Discussion for Contribution Points of the Paper

The paper proposed a mechanical design analysis of SEA-driven robot leg and controller design to realize the SLIP dynamics. The discussions for the contribution of this paper are categorized as shown in the following subsections.

#### 4.1.1. Advantage of SEA-Driven Robot

The SEA, utilized in this paper, is one emerging actuator system in the robotics field. The benefits of the SEA are the torque control capability based on a spring deflection measurement. This paper has analyzed the dynamics of SEA and designed the DOB-based torque controller based on the dynamics. The robustness has been examined with regards to the variation of the load inertia. This is a significant feature when the SEA is used in multi-joint robots such as the robot leg in this paper, since the inertia varies continuously under the dynamic condition. The other advantageous feature of SEA is that the SEA can be used as an identification tool with a specific mechanical design such as the biarticular mechanism in this paper. SEA has various output states; namely, it is a multi-output system. In this paper, the motor side position control has been utilized to identify the physical parameters of the robot leg. This enables the dynamic parameter identification without any extra-measurement of link mass or center of mass, and it allows an accurate dynamics-based control including gravity compensation, inertia decoupling and inertia modulation.

#### 4.1.2. Benefits of Biarticular Mechanism and Rotating Workspace Coordination in Terms of SLIP Dynamics Realization

This paper has developed the SEA-driven articulated robotic leg with the biarticular actuation mechanism taking advantage of biarticular muscles in their design. The design has complemented the robot actuator coordination and human musculoskeletal system. In addition, the RW, which is suitable to the coordinate system of the SLIP model, has been utilized and analyzed in the operation of the biarticular actuator-coordinated robot to satisfy the requirement for human-like locomotion.

Taking advantage of biarticular actuator coordination, a systematic strategy was shown to compensate for the complex dynamic behavior of the robot leg in the Rotating Workspace within bio-inspired coordination. Based on this, the RWHC has been developed to enable the biarticular mechanism to realize SLIP behavior robustly.

#### 4.1.3. Control Performance of the Proposed Hybrid Control in Rotating Workspace

The experiments have verified the performance of the proposed Rotating Workspace Hybrid Control: impedance control performance of the robotic leg in the interactive direction and robust position tracking in the tangential direction, and robustness of radial directional impedance control during high accuracy tangential control. It has been shown through the experimental results that the developed SEA-driven robot leg with the proposed Rotating Workspace Hybrid Control including two-axes controllers could realize the SLIP dynamics robustly.

The dynamic performance depends on the body state; hence, this approach cannot be generalized for every legged robot. For example, the dynamic reaction of the body of quadrupeds and bipeds are totally different. Therefore, the theory and experiments have been analyzed and performed in a given fixed body condition. In order to extend the proposed approach, the dynamics with the unfixed body condition should be considered and compensated, even though the controller performs well in a given condition.

### 4.2. Conclusions and Future Work

Human locomotion leads to a simple result of moving the body position; however, the dynamical operation, which is realized in the human leg, is complicated. In order to analyze human locomotion, an analytical tool that can make descriptions from the viewpoint of being outside of human motions as simple as possible is required, rather than a complex neuromuscular analysis approach that considers all the muscles' coordination from a vantage point inside the human body. Robotics, which has been developed mainly from the perspectives of engineering, now starts to turn to humans and nature. The bio-inspired actuation and coordination systems and their insights obtained through the observation on the human and nature will provide a new perspective to establish fundamentals for bio-inspired robotics.

In this article, biarticular actuator coordination for designing, analyzing and controlling robotic systems has been investigated. This paper showed a serial procedure to realize the SLIP dynamics for the articulated robot. Based on the realized SLIP dynamics, the foundation for extending the robot leg to various locomotion such as running, gaiting and so on has been discussed. Such mechanical design and control technology is an underlying technology that can be applied to bipedal and quadrupedal walking robots. Therefore, the future work of this research is to utilize the proposed technique in hopping and walking motions of the robot leg and to extend the technique to a quadruped robot.

## Author Contributions

CL contributed to literature writing and review, fabrication of the robot, controller design and experiments. SO developed workspace coordinates and supervised the project overall, and contributed to data analysis and writing the paper.

### Conflict of Interest Statement

The authors declare that the research was conducted in the absence of any commercial or financial relationships that could be construed as a potential conflict of interest.

## Abbreviations

DOB: Disturbance Observer
SEA: Series Elastic Actuator
SLIP: Spring Loaded Inverted Pendulum.
